# Identification of a Five-Gene Signature Derived From MYCN Amplification and Establishment of a Nomogram for Predicting the Prognosis of Neuroblastoma

**DOI:** 10.3389/fmolb.2021.769661

**Published:** 2021-12-07

**Authors:** Yuren Xia, Xin Li, Xiangdong Tian, Qiang Zhao

**Affiliations:** ^1^ National Clinical Research Center for Cancer, Key Laboratory of Cancer Prevention and Therapy, Tianjin’s Clinical Research Center for Cancer, Tianjin Medical University Cancer Institute and Hospital, Tianjin, China; ^2^ Tianjin Cancer Hospital Airport Hospital, Tianjin, China

**Keywords:** neuroblastoma, gene signature, MYCN amplification, nomogram, prognosis

## Abstract

**Background:** Neuroblastoma (NB), the most common solid tumor in children, exhibits vastly different genomic abnormalities and clinical behaviors. While significant progress has been made on the research of relations between clinical manifestations and genetic abnormalities, it remains a major challenge to predict the prognosis of patients to facilitate personalized treatments.

**Materials and Methods:** Six data sets of gene expression and related clinical data were downloaded from the Gene Expression Omnibus (GEO) database, ArrayExpress database, and Therapeutically Applicable Research to Generate Effective Treatments (TARGET) database. According to the presence or absence of MYCN amplification, patients were divided into two groups. Differentially expressed genes (DEGs) were identified between the two groups. Enrichment analyses of these DEGs were performed to dig further into the molecular mechanism of NB. Stepwise Cox regression analyses were used to establish a five-gene prognostic signature whose predictive performance was further evaluated by external validation. Multivariate Cox regression analyses were used to explore independent prognostic factors for NB. The relevance of immunity was evaluated by using algorithms, and a nomogram was constructed.

**Results:** A five-gene signature comprising CPLX3, GDPD5, SPAG6, NXPH1, and AHI1 was established. The five-gene signature had good performance in predicting survival and was demonstrated to be superior to International Neuroblastoma Staging System (INSS) staging and the MYCN amplification status. Finally, a nomogram based on the five-gene signature was established, and its clinical efficacy was demonstrated.

**Conclusion:** Collectively, our study developed a novel five-gene signature and successfully built a prognostic nomogram that accurately predicted survival in NB. The findings presented here could help to stratify patients into subgroups and determine the optimal individualized therapy.

## Introduction

Neuroblastoma (NB) is the most common extracranial solid tumor in children, with the highest incidence and mortality in infancy, accounting for approximately 8–10% of pediatric malignancies (Li et al., 2008; Marshall et al., 2014). It is a highly heterogeneous disease characterized by diverse clinical manifestations ranging from spontaneous regression to progression with therapy resistance. Based on clinical and molecular patterns, NB patients can be classified into low-risk, intermediate-risk, and high-risk groups (Cohn et al., 2009). Although the prognosis of NB has significantly improved over the past decade with the development of multiple treatment methods and the application of immunotherapy targeting NB-specific antigens, such as GD2 (Casey and Cheung, 2020), less than 40% of high-risk NB patients achieve long-term survival (Pinto et al., 2015). Given the significantly divergent outcomes among these patients, an effective prediction model is needed so that effective management and therapy can be tailored to diverse subgroups of NB patients.

There is increasing evidence that several genomic alterations, such as gain of chromosome 17q, hemizygous deletions of 1p and 11q, and MYCN gene amplification, are powerful prognostic markers that are strongly associated with clinical outcomes (Janoueix-Lerosey et al., 2009; Maris, 2010; Schleiermacher et al., 2012). Among these factors, MYCN amplification is one of the earliest biomarkers discovered in NB and is still considered one of the most reliable predictors of aggressive clinical behavior (Park et al., 2013; Campbell et al., 2017). Augmented MYCN is detected in 20–30% of all NB cases, and the overall survival rate for this group remains less than 50% (Huang and Weiss, 2013). Although MYCN plays a pivotal role in NB development, its application as a therapeutic target remains challenging. Although much research has been done on targeting MYCN-related factors, new target genes are still needed to effectively treat this aggressive and heterogeneous disease (Puissant et al., 2013; Gustafson et al., 2014; Huang et al., 2019). Due to the rapid development of high-throughput sequencing, multigene signatures have been shown in many studies to be more effective at predicting prognosis than conventional biomarkers. The genes discovered by bioinformatic approaches can also provide insights into cancer progression and identify pathway profiles involved in tumorigenesis. Several NB prognostic models have been made based on sequencing data (Vermeulen et al., 2009; De Preter et al., 2010; He et al., 2020; Wang et al., 2020). A deeper dive into public data sets may reveal other genes associated with prognosis and new potential therapeutic targets.

Herein, we aimed to build a prognostic signature that could predict the prognosis of NB patients more accurately than traditional clinical factors. We systematically analyzed multiple public NB data sets to identify differentially expressed genes (DEGs) based on MYCN amplification. Then, we successfully established a prognostic five-gene risk prediction model that can accurately predict the outcome of NB. Simultaneously, we performed preliminary validation of the pivotal DEGs using clinical samples. A prognostic nomogram was finally designed based on the gene signature and clinical factors, and this nomogram will help physicians develop more customized treatment plans for different subgroups of NB patients.

## Materials and Methods

### Data Set

NB gene expression data sets and related clinical information were downloaded from the Gene Expression Omnibus (GEO) under accession numbers GSE45547 (Kocak et al., *n* = 649), GSE49710 (Zhang et al., *n* = 498), GSE73517 (Henrich et al., *n* = 105), and GSE120559 (Fischer et al., *n* = 208) and ArrayExpress under accession number E-MTAB-8248(Christoph et al., *n* = 223). The four GEO data sets were used for DEG analysis. The Therapeutically Applicable Research to Generate Effective Treatments (TARGET) NB gene expression data sets and related clinical information were downloaded using cBioPortalData in the R client. The TARGET data sets were used for prognostic gene signature prediction. E-MTAB-8248 and GSE49710 with complete follow-up information were used for prognostic signature validation.

### Differentially Expressed Gene Analysis

Patients were stratified into MYCN amplification and MYCN nonamplification groups according to the clinical information provided in the data sets. In the case of a gene with multiple probes matching it, its expression level was calculated by taking the highest expression value. DEGs were explored between two groups using the “limma” R package (Ritchie et al., 2015). The DEG cutoff was set as |log2 (fold-change) | > 1 and adjusted *p*-value < 0.05. The volcano plots were created using the R package Enhanced Volcano. The DEGs identified from the four data sets from GEO were further analyzed using the robust rank aggregation (RRA) method-based R package “RobustRankAggreg” (Kolde et al., 2012). ***p*** *< 0*.*05* was considered statistically significant in this study.

### Enrichment of Differentially Expressed Genes

DEG functional enrichment analysis, including Gene Ontology (GO) and Kyoto Encyclopedia of Genes and Genomes (KEGG) analysis, was carried out using the “clusterProfiler” R package (Yu et al., 2012).

### Protein–Protein Interaction Network of the Differentially Expressed Genes

To investigate the potential hub genes among the DEGs, we constructed a protein–protein interaction (PPI) network of DEGs using the Search Tool for the Retrieval of Interacting Genes/Proteins (STRING) online database (https://string-db.org, Version: 11.0) with an interaction score of 0.90 and Cytoscape software (https://cytoscape.org, Version: 3.8.2) (Smoot et al., 2011; Szklarczyk et al., 2019). The hub nodes were identified by the maximal clique centrality (MCC) method in the CytoHubba plugin. Densely connected clusters were identified in the MCODE plugin.

### Establishment of the Prognostic Gene Signature

The TARGET-NBL mRNA expression data were used for prognostic gene signature establishment. The DEGs identified from the GEO data sets were used for least absolute shrinkage and selection operator (LASSO)-penalized Cox regression analysis in the “glmnet” package in R. Subsequently, multivariate Cox regression analysis was used to further reduce the number of DEGs for model construction. Finally, a prognostic signature of NB was constructed. The risk score was calculated by multiplying the mRNA expression level by the regression coefficient for each gene. According to the median risk score, patients were divided into low-risk and high-risk groups. The predictive performance of the signature was evaluated according to Kaplan–Meier (KM) analysis, the area under the curve (AUC) of the receiver operating characteristic (ROC) curve and Harrell’s concordance index (C-index). The GSE49710 and E-MTAB-8248 data sets were used for external data set validation.

### Assessment of Tumor Immunity

The tumor microenvironment of each NB sample was evaluated via the Estimation of STromal and Immune cells in MAlignant Tumor tissues using the Expression data (ESTIMATE) algorithm. ESTIMATE is a sophisticated algorithm that estimates the degree of infiltration of tumor cells and determines the stromal score, immune score, and estimate score (Yoshihara et al., 2013). The degree of immune cell infiltration was estimated via Cell Type Identification by Estimating Relative Subsets of RNA Transcripts (CIBERSORT), which is a deconvolution algorithm for analyzing the expression matrix of immune cell subtypes (Newman et al., 2015).

### Preparation of Tissue Samples

The study was permitted by the Ethics Committee of the Tianjin Medical University Cancer Institute and Hospital (Approval No. E20210027). Five MYCN-amplified NB samples and five MYCN-nonamplified NB samples were harvested from NB patients at Tianjin Medical University Cancer Institute and Hospital. All patients were in International Neuroblastoma Staging System (INSS) stage 3 and stage 4. The tissue samples were stored at −80°C until use. All patients provided written informed consent.

### Quantitative Real-Time PCR

Total RNA was extracted from NB tissues using the TRIzol reagent (Ambion, United States). Complementary DNA (cDNA) was synthesized using a quantitative real-time polymerase chain reaction (RT-PCR) kit (Takara, Japan). Quantitative RT-PCR was performed using an miScript PCR system (QIAGEN) according to the manufacturer’s instructions. The qRT-PCR data were assessed via the 2-ΔΔCt method with β-actin as the control. The primer sequences are summarized in Supplementary Table S1.

### Independent Prognostic Parameters of Neuroblastoma Identification

To identify independent prognostic factors in the TARGET data set, univariate analyses were performed on the five-gene signature as well as clinical and pathological parameters, including sex, age, MYCN status, tumor histology, race, and tumor stage. Statistically significant parameters were further included in multivariate Cox regression analyses and nomogram construction.

### Nomogram Construction

A prognostic nomogram including all independent prognostic factors was constructed to predict the overall survival of NB patients in the TARGET cohort. The predictive performance of the nomogram was evaluated according to KM analysis, the AUC of the ROC curve, and the C-index.

### Statistical Analysis

We performed all the statistical analyses with RStudio (1.1.463). Mann–Whitney U-tests and Kruskal–Wallis tests were used to compare two groups. Survival curves were plotted and compared using the KM method and log-rank analysis. Survival data were analyzed via univariate and multivariate Cox regression analysis. A *p* value <0.05 was considered to indicate statistical significance.

## Results

### Identification of Differentially Expressed Genes

**FIGURE 1:**
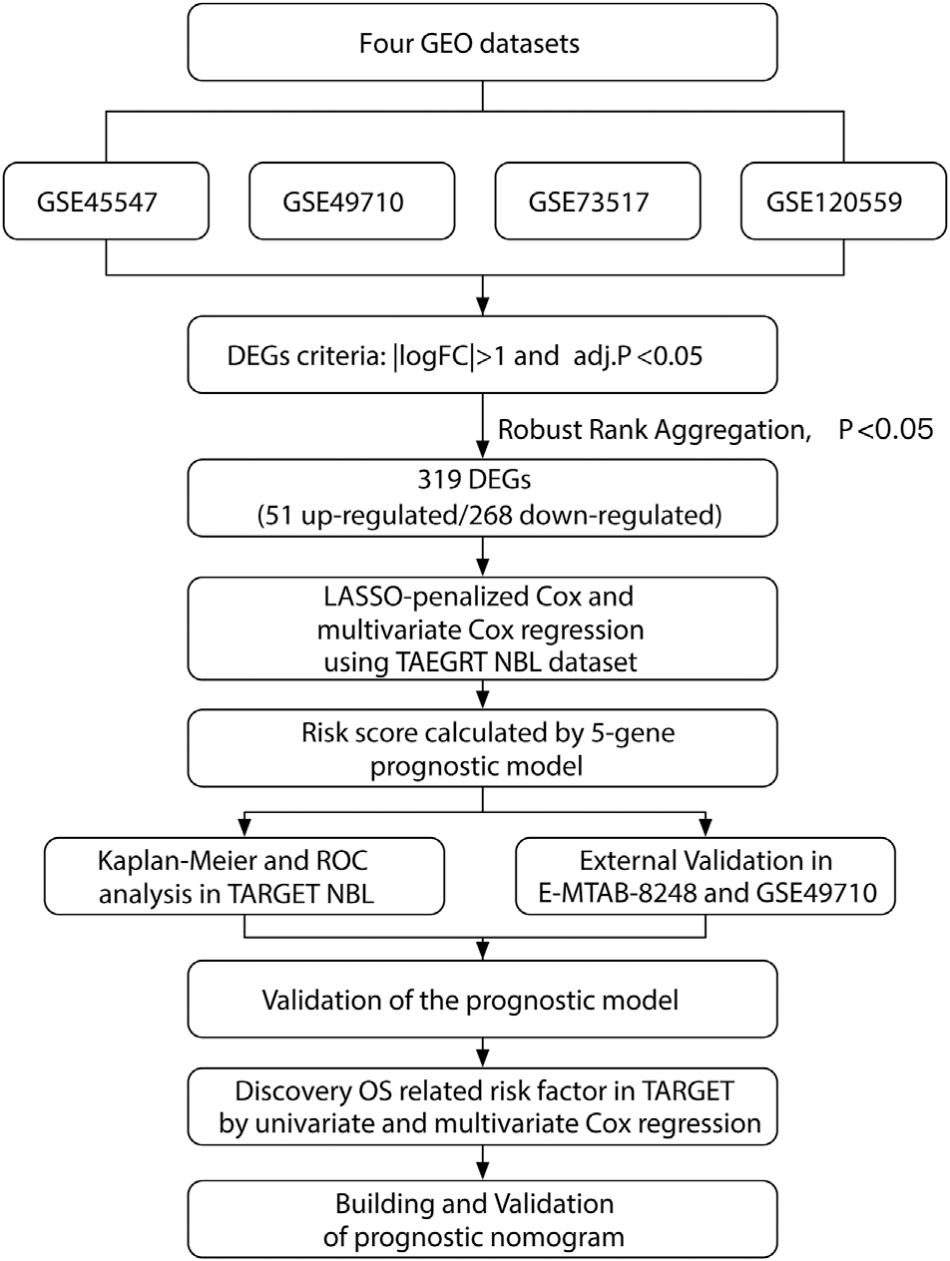
Flowchart illustrating the research design.

**FIGURE 2:**
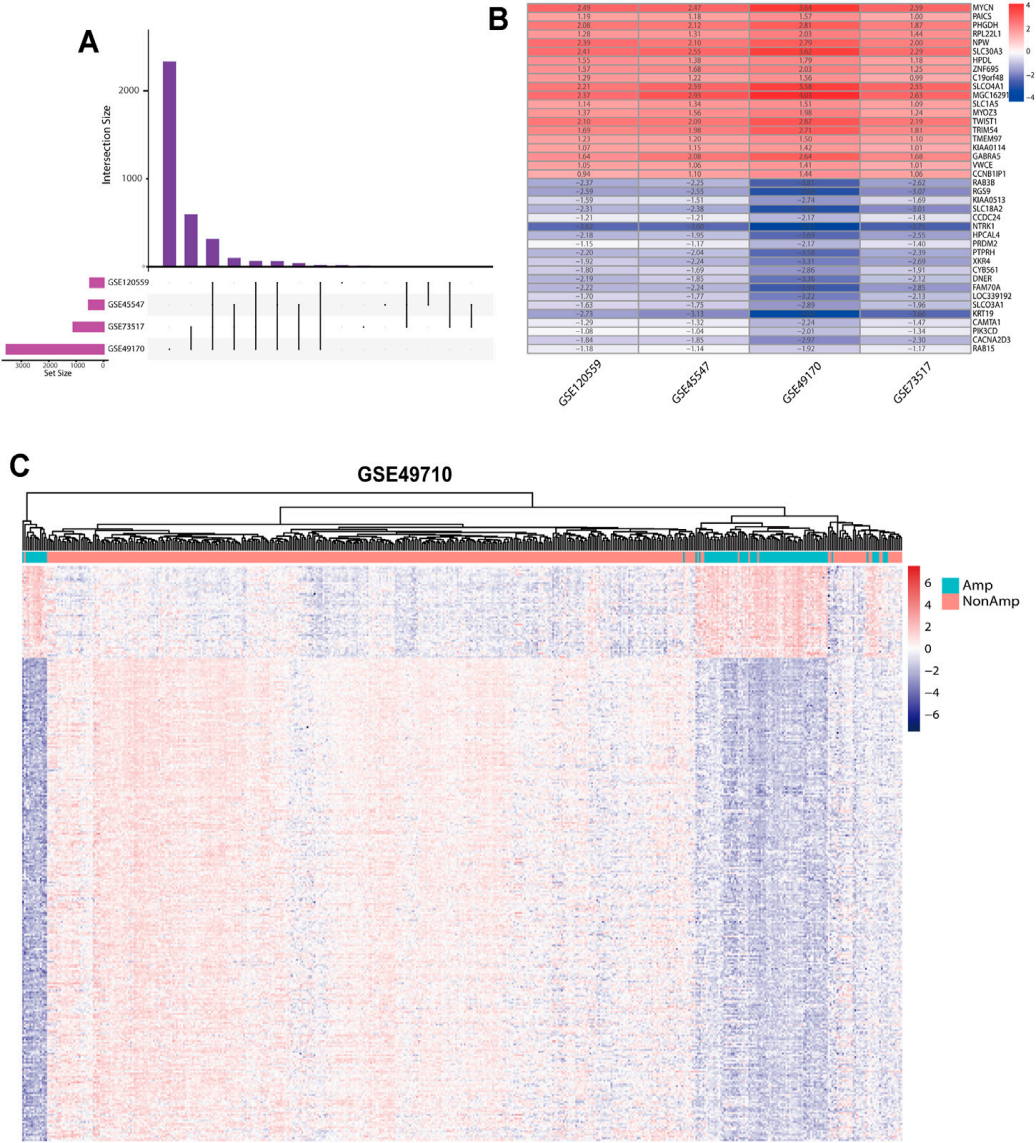
DEGs between the MYCN amplification and nonamplification groups. **(A)** Venn diagram of the DEGs obtained from four GEO data sets. **(B)** Heatmap of the top 20 upregulated and downregulated DEGs derived from the GEO data sets. Red represents upregulated DEGs, and blue represents downregulated DEGs. **(C)** Heatmap of the 319 DEGs after integrated analysis in the GSE49710 data set.

Our study was performed based on the flow chart illustrated in Figure 1. Detailed information on the GEO, TARGET, and ArrayExpress data sets in this project is shown in Supplementary Table S2. The DEG criteria were set as |logFC|>1 and *adj*. *p* < 0.05 in all data sets. Four sets of DEGs (GSE45547, GSE49710, GSE73517, and GSE120559) comprising 765 (225 up/315 down), 3538 (284 up/3254 down), 1100 (117 up/983 down), and 494 (184 up/310 down) DEGs each were identified between the MYCN amplification and nonamplification groups (Figure 2A and Supplementary Figure S1A–D). Through the robust rank aggregation (RRA) method, DEGs derived from four data sets were integrated and analyzed. A total of 319 DEGs, including 51 upregulated and 268 downregulated genes, were identified (Supplementary Table S3). Figure 2B depicts the top 20 genes that were identified as upregulated and downregulated by using the RRA method. In clustering analyses, a marked difference in DEG expression appeared between the MYCN amplification and nonamplification groups (Figure 2C and Supplementary Figure S2A–C).

### Enrichment Analysis of Differentially Expressed Genes

**FIGURE 3:**
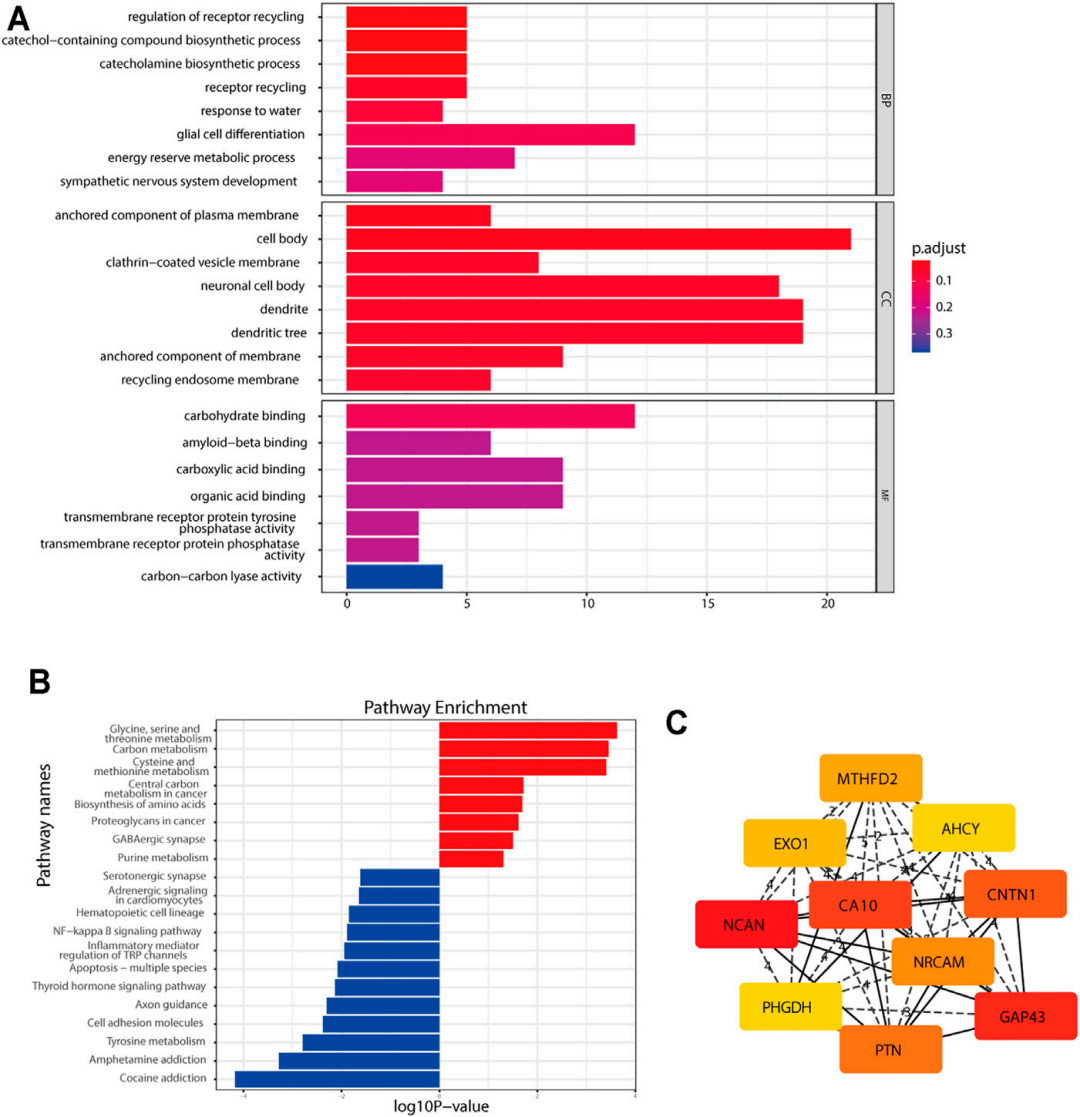
Functional enrichment analysis of the DEGs. **(A)** GO analysis of the DEGs. **(B)** KEGG pathway enrichment analysis of the DEGs. **(C)** Top 10 hub genes found by the CytoHubba plugin in Cytoscape software through the MCC method.

GO and KEGG analyses were performed to determine the potential functions of DEGs with the clusterProfiler R package (Supplementary Tables S4, S5). The DEGs were significantly enriched in biological processes, such as glial cell differentiation, the receptor recycling process, the catecholamine biosynthetic process, sympathetic nervous system development, and central nervous system neuron differentiation; cellular components, such as neuronal cell bodies and dendrites; and molecular functions, such as carbohydrate binding (Figure 3A). In the KEGG pathway analysis, DEGs were shown to be involved in the amino acid metabolism and cell adhesion (Figure 3B). To identify gene interactions, a PPI network containing 168 nodes and 312 interactions was constructed. Figure 3C shows the top 10 candidate hub genes identified in this network. Module analysis identified the top two highest-scoring clustering modules identified by using module analysis (Supplementary Figures S3A, C). According to functional enrichment analysis, module 1 had a strong association with synapse formation and the differentiation of glia (Supplementary Figure S3B). Module 2 showed a significant correlation with the amino acid metabolism process and DNA replication, indicating its involvement in the tumor-related metabolism and DNA replication (Supplementary Figure S3D). The above results of the PPI network indicated that the DEGs were involved in the NB cell differentiation and metabolism.

### Construction of a Five-Gene Prognostic Signature

**FIGURE 4:**
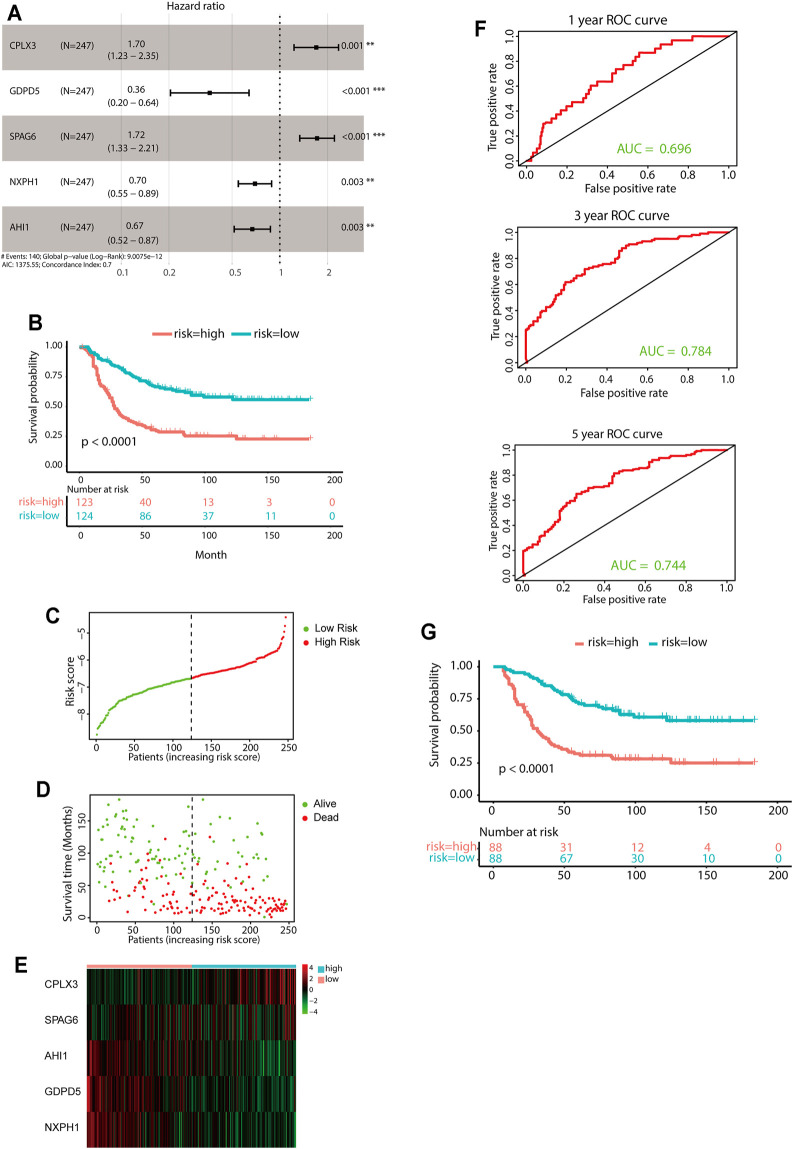
Construction of the predictive five-gene signature. **(A)** Prognostic effect of the five-gene signature derived from stepwise Cox regression survival analysis for overall survival in the TARGET data set. **p < 0.01, ***p < 0.001. **(B)** KM curves for overall survival of the two risk groups derived from the five-gene signature in the TARGET data set. The *p*-value was calculated by the log-rank test. **(C-E)** Distribution of the risk score **(C)**, the associated survival data **(D)**, and the five-gene mRNA expression **(E)** in the TARGET data set. **(F)** ROC curves for 1-year, 3-year, and 5-year overall survival predictions for the five-gene signature. **(G)** KM survival curves of the five-gene signature in the MYCN nonamplification group.

A total of 247 patients from the TARGET-NBL gene expression data sets were included for subsequent survival analyses. The detailed characteristics of the 247 patients are shown in Supplementary Table S6. LASSO-penalized Cox regression analysis identified 35 DEGs significantly correlated with survival (lambda = 0.05, Supplementary Figure S4). After optimization of 35 DEGs by the multivariate Cox regression method, a prognostic signature consisting of five genes, including CPLX3, GDPD5, SPAG6, NXPH1, and AHI1, was constructed. The downregulated genes GDPD5, NXPH1, and AHI1 were identified as tumor suppressors, while the upregulated genes CPLX3 and SPAG6 were regarded as oncogenes (Figure 4A). The following equation was used for risk score calculations:

Risk score = [(0.5292 × expression value of CPLX3) + [(−1.0179) × expression value of GDPD5] + [(0.5403) × expression value of SPAG6] + [(−0.3607) × expression value of NXPH1] + [(−0.3993) × expression value of AHI1].

The median value of the risk score was set as the cutoff value. Patients from the TARGET-NBL data set were categorized into two groups. The KM survival curve revealed significantly favorable outcomes in the low-risk-score group (log-rank *p* < 0.0001, Figure 4B). The high-risk group had poor prognosis (Figures 4C–E). Evaluation of the five-gene prediction model was carried out using time-dependent ROC curves and the C-index. The AUCs for the 1-, 3-, and 5-year survival were 0.696, 0.784, and 0.744, respectively (Figure 4F), and the C-index of the gene signature was 0.697 (95% CI: 0.656–0.738). Patients without MYCN amplification were further stratified into two groups based on the median risk score, and the KM survival curves showed a significant difference in prognosis between the high- and low-risk groups (log-rank *p* < 0.0001, Figure 4G). In general, the five-gene signature was highly accurate for predicting prognosis.

### Validation of the Gene Signature

**FIGURE 5:**
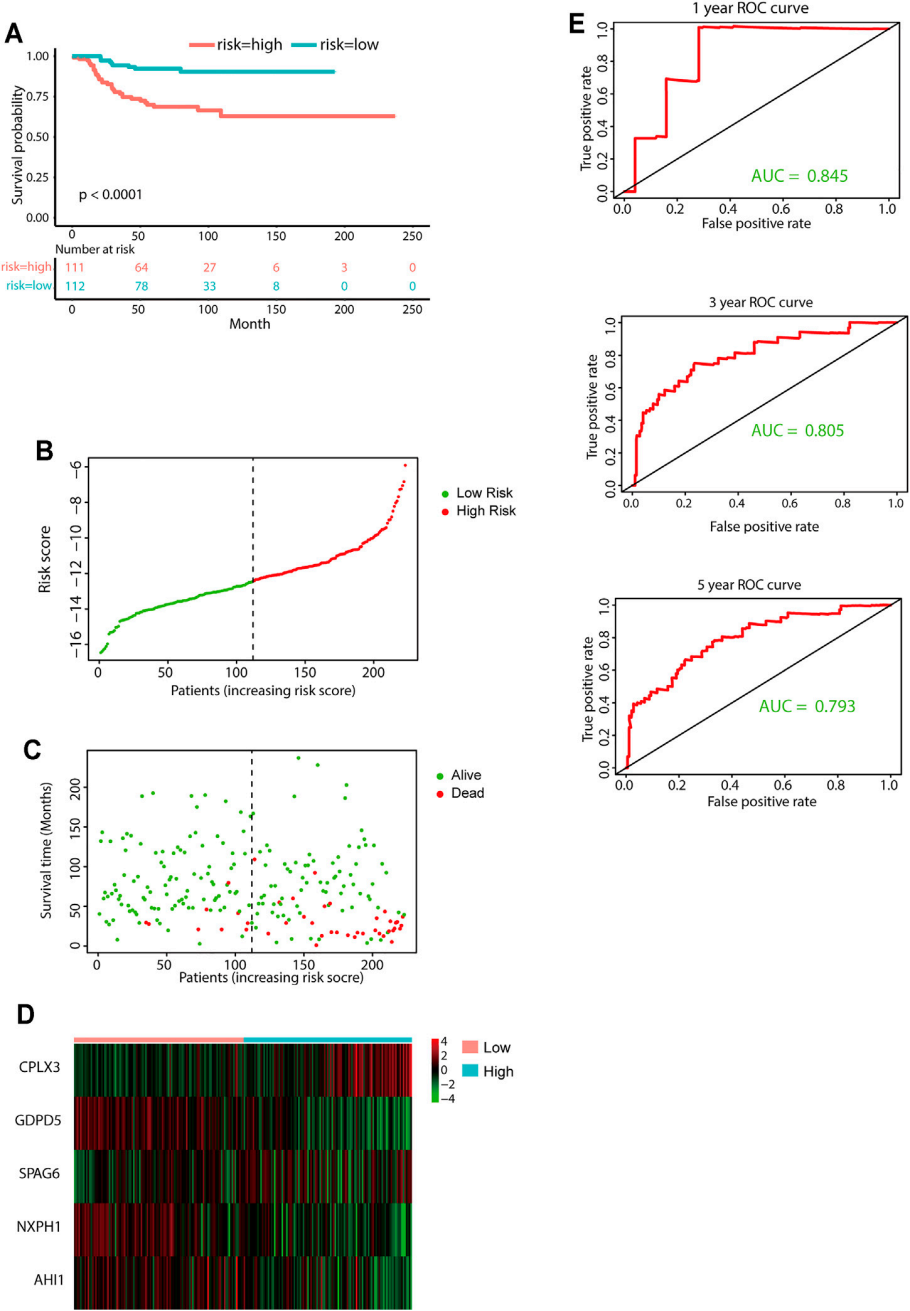
External validation of the five-gene signature. **(A)** KM curves for overall survival of the two risk groups derived from the five-gene signature in the E-MTAB-8248 data set. The *p*-value was calculated by the log-rank test. **(B-D)** Distribution of the risk score **(B)**, the associated survival data **(C)**, and the five-gene mRNA expression **(D)** in the E-MTAB-8248 data set. **(E)** ROC curves for 1-year, 3-year, and 5-year overall survival predictions for the five-gene signature in the E-MTAB-8248 data set.

The E-MTAB-8248 and GSE49710 data sets, which contain overall survival data, were utilized to validate the performance of the prognostic model (Figure 5; Supplementary Figure S5). Calculations of the risk score were made using the same equation for all patients. Based on the median risk score, the patients were divided into low- and high-risk groups. The high-risk group had markedly worse outcomes than the low-risk group in both validation cohorts (Figures 5A–D and Supplementary Figures S5A–D). Further evaluation of the predictive accuracy was conducted using time-dependent ROC curve analysis and the C-index. The five-gene signature performed well in both short-term and long-term survival prediction (Figure 5E and Supplementary Figure S5E).

### Prognostic Value Comparison Between the Five-Gene Signature, INSS Staging, and MYCN Amplification Status

**FIGURE 6:**
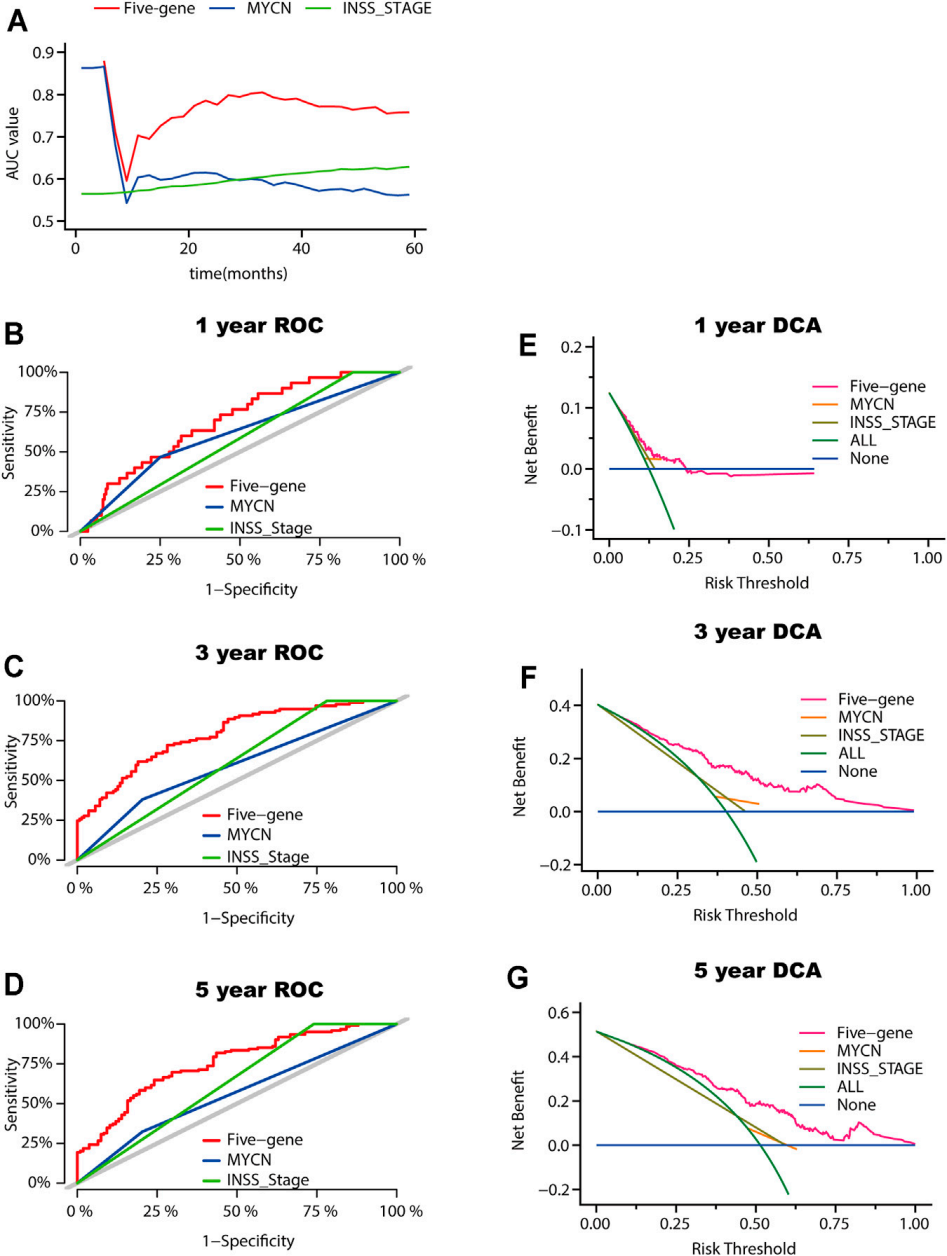
Model comparison between the five-gene signature, the INSS stage, and the MYCN status. **(A)** AUC of the five-gene signature, the INSS stage, and the MYCN status. **(B-D)** ROC curves of the five-gene signature of the INSS stage model and MYCN status for 1-year **(B)**, 3-year **(C)**, and 5-year **(D)** overall survival predictions. **(E-G)** DCA curves depicting the standardized net benefit of the five-gene signature for INSS stage and MYCN status at 1 year **(E)**, 3 years **(F)**, and 5 years **(G)**.

INSS stage and MYCN status are both commonly used clinicopathologic factors for predicting NB prognosis. We next compared the predictive ability between the five-gene signature and INSS staging as well as MYCN amplification status. The AUCs for 1-, 3-, and 5-year overall survival predictions for the risk scores were 0.696, 0.784, and 0.744, respectively. The AUCs for 1-, 2-, and 3-year overall survival predictions for INSS staging and MYCN status were 0.573, 0.609, and 0.630 and 0.608, 0.588, and 0.560, respectively (Figures 6A–D). Decision curve analyses (DCAs) for 1-, 3-, and 5-year overall survival predictions showed better efficiency for the five-gene signature than the INSS staging and MYCN status. The areas under the decision curve of the five-gene signature were 0.00975, 0.13147, and 0.17676 for 1-, 3-, and 5-year survival, respectively, while those of INSS staging and MYCN status were 0.00880, 0.09328, and 0.15217 and 0.00086, 0.00595, and 0.00478, respectively (Figures 6E–G). Overall, the five-gene signature was more effective in predicting the survival of NB.

### Correlations of Clinical Pathology and Tumor Immune Cell Infiltration With the Five-Gene Signature

**FIGURE 7:**
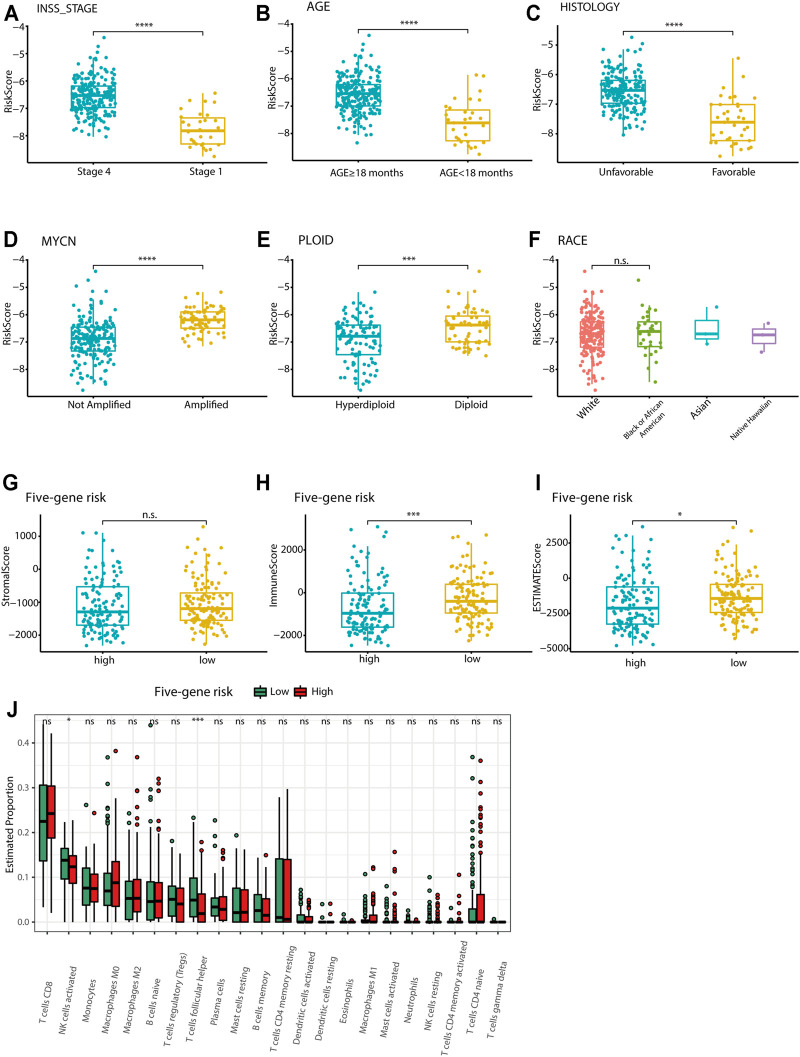
Association of the five-gene signature with clinicopathological parameters and tumor immune components. **(A-F)** Box plots of the distribution of the five-gene risk score between different INSS stage groups **(A)**, between the age ≥18 months and age <18 months groups **(B)**, between the favorable and unfavorable histology groups **(C)**, between the MYCN-amplified and MYCN-nonamplified groups **(D)**, between the hyperdiploid and diploid groups **(E)**, and between different race groups **(F)** in the TARGET data set. ***p < 0.001, ****p < 0.0001, n. s. not significant, two-sided unpaired Wilcoxon test. **(G-I)** Box plots of the distribution of the stromal score **(G)**, immune score **(H)**, and estimate score **(I)** calculated by the ESTIMATE algorithm between the high-risk and low-risk groups. *p < 0.05, ***p < 0.001, n. s. not significant, two-sided unpaired Wilcoxon test. **(J)** Box plots of the distribution of the cell proportions calculated by the CIBERSORT algorithm of immune cells between the high-risk and low-risk groups. *p < 0.05, ***p < 0.001, n. s. not significant, two-sided unpaired Wilcoxon test.

Relationships between the five-gene signature risk score and the clinical characteristics of NB patients, including INSS stage, age, MYCN status, tumor histology, tumor ploidy, and race, were analyzed in TARGET data sets. In terms of INSS stage, a higher risk score was observed in patients in stage IV than in those in stage I in the TARGET data set (Figure 7A). The five-gene risk score increased from stage 1 to stage 4, except for stage 4S in GSE49710 and E-MTAB-8248 (Supplementary Figures S6A, D). Patients aged ≥18 months or with unfavorable histology, MYCN amplification, and diploid tumors had higher five-gene risk scores (Figures 7B–E). The risk scores showed no difference according to race (Figure 7F). Two extra data sets also validated that the MYCN amplification group had higher five-gene risk scores (Supplementary Figures S6B, S6E). Analysis of the GSE49710 data set revealed that patients with higher five-gene risk scores tended to experience disease progression (Supplementary Figure S6F).

The ESTIMATE algorithm was used to investigate the correlation between the five-gene signature and immune cell infiltration. The stromal score, immune score, and estimate score were calculated for the TARGET data set. However, the stromal score showed no difference between the high-risk and low-risk groups. The immune score and estimate scores were significantly higher in the low-risk group, indicating the enrichment of infiltrating immune cells in the low-risk group (*p* < 0.05; Figures 7G–I). Further analysis by the CIBERSORT algorithm showed that the five-gene low-risk group had higher levels of follicular helper T cell and activated NK cell infiltration (Figure 7J). The E-MTAB-8248 and GSE49710 data sets also validated the significant difference between the two groups in immune score and estimate score (Supplementary Figures S6F–J). However, the results of the CIBERSORT algorithm in both validation sets were different from those of the training set, where the levels of CD8^+^ T cells and M2-type macrophages were found to be much higher in the low-risk group (Supplementary Figure S7).

### Validation of the Five Differentially Expressed Genes Used for the Gene Signature

The mRNA expression levels of the five pivotal genes were measured in five MYCN-amplified NB tissues and five MYCN-nonamplified tissues by qRT-PCR. This result was consistent with the above DEG analysis between the MYCN amplification and MYCN nonamplification groups in the four data sets (Supplementary Table S3). As shown in Supplementary Figure S8, compared with MYCN-nonamplified tissues, the expression level of CPLX3 was elevated in MYCN-amplified tissues. The other four genes were found to be downregulated in the MYCN-amplified group.

### Evaluation of Independent Prognostic Factors in Neuroblastoma

A total of 220 patients from the TARGET data set with sufficient clinical information were included in the analysis. Overall survival-related independent prognostic factors were identified through stepwise Cox regression analysis. The univariate analysis showed that the five-gene risk score (*p* < 0.0001), tumor histology (*p* < 0.0001), age (*p* = 0.00065), and MYCN status (*p* = 0.024) were associated with the prognosis of NB (Supplementary Table S7). The five-gene risk score and age were independently associated with overall survival according to multivariate Cox regression analysis (Supplementary Table S8).

### Establishment and Validation of a Nomogram

**FIGURE 8:**
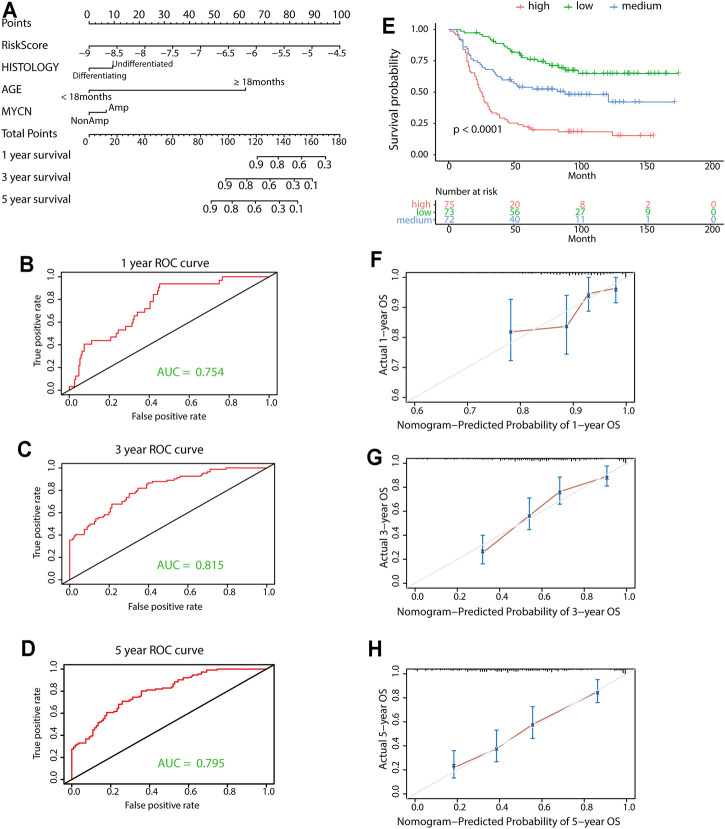
Construction and validation of the nomogram model. **(A)** Nomogram model for 1-year, 3-year, and 5-year overall survival probability predictions in the TARGET data set. **(B-D)** Time-dependent ROC curves for survival predictions for the nomogram. **(E)** KM survival curves of the nomogram. Patients from the TARGET data set were classified into three groups based on the tertile point calculated by the nomogram model. **(F-H)** Calibration plot for internal validation of the nomogram. The *Y*-axis represents the actual overall survival, while the *X*-axis represents the predicted overall survival.

A prognostic nomogram was built for predicting overall survival based on the stepwise Cox regression analysis results. Risk score, age, tumor histology, and MYCN status were parameters included in the nomogram (Figure 8A). The AUCs of the 1-, 3-, and 5-year overall survival predictions for the nomogram model were 0.754, 0.815, and 0.795, respectively (Figures 8B–D). The C-index of the risk score was 0.717 (95% CI: 0.674–0.760). According to the scores of the nomogram model, the patients were divided into three equal groups. There was good discrimination between these groups in the KM plots (*p* < 0.0001) (Figure 8E). Calibration plots at 1, 3, and 5 years showed that the nomogram correctly predicted the NB patient’s overall survival (Figures 8F–H).

## Discussion

In the current study, we developed a novel multigene signature that showed better predictive power than conventional biomarkers. At present, there is still an urgent need for new effective stratification systems and treatment targets for NB. Although it has been well established that MYCN amplification affects the prognosis of NB (Brodeur et al., 1984; Seeger et al., 1985), it remains challenging to target this oncogene in clinical treatment. The development of the sequencing technology allows us to deeply explore the molecular mechanism by which MYCN amplification affects prognosis at the whole-genome level. Relevant NB data were obtained from multiple data sets, and patients were grouped based on MYCN status. We identified 319 DEGs, including 51 upregulated and 268 downregulated genes. GO and KEGG enrichment analyses of these DEGs were performed to further investigate the molecular mechanism of NB. The DEGs were shown to be significantly enriched in biological processes linked to the occurrence and development of NB, such as glial cell differentiation, the catecholamine biosynthetic process, and sympathetic nervous system development. The disialoganglioside GD2, a unique carbohydrate antigen, is one of the most attractive immune surface targets for NB. GD2 is uniformly expressed by NB cells, which makes GD2 an ideal therapeutic target for NB (Suzuki and Cheung, 2015; Sait and Modak, 2017). According to GO analysis, the DEGs in our study were significantly enriched in molecular functions involved in carbohydrate binding. Among these DEGs, there may be potential regulatory genes associated with GD2 immunotherapy. KEGG pathway analysis showed that the DEGs were mainly enriched in cancer-related pathways, such as the amino acid metabolism and cell adhesion processes. Cancer cells require an abundant supply of amino acids to sustain their growth (Vettore et al., 2020), and an increased amino acid metabolism has been observed in chemoresistant NB cell lines (Gunda et al., 2020). The cancer metastatic cascade is dependent on the loss of adhesion between cells, resulting in the dissociation of the cell from the primary tumor (Martin and Jiang, 2009). The subsequent PPI network results further confirmed that these DEGs participated in the NB cell differentiation and metabolism.

We further screened 35 DEGs that significantly affected survival. Afterward, a risk prediction model consisting of five genes (GDPD5, NXPH1, AHI1, CPLX3, and SPAG6) was established. The upregulated genes CPLX3 and SPAG6 appeared to be associated with poor survival, whereas the downregulated genes GDPD5, NXPH1, and AHI1 were identified as tumor suppressors. The outcome was significantly different between NB patients with high and low risk scores. Additionally, the correlation between NB clinical features and the five-gene signature was investigated. Many known risk factors for NB that affect prognosis were shown to be related to a high risk score. Furthermore, the risk score for patients in each INSS stage increased from stage 1 to stage 4, except for stage 4S, which often has a favorable outcome (Kawano et al., 2021). Collectively, these findings suggest that patients with high risk scores exhibit more aggressive features than those with low risk scores. Subsequent external validation further confirmed that the five-gene signature could accurately predict survival in NB patients. To assess its clinical efficacy, we compared the gene signature with INSS staging and MYCN status, which are commonly used in predicting outcome in the clinic. The AUC and DCA curves also confirmed that the five-gene signature had a better prognosis prediction than the INSS stage and MYCN status, both in the short term and in the long term. In the MYCN nonamplification group, the gene model was still able to provide a good indication of the prognosis, which may aid physicians in evaluating prognosis and optimizing treatment options for this cohort of patients.

Due to the complex biological and clinical characteristics of NB, conventional staging systems may be difficult to utilize for calculating tumor risk in an accurate manner. To determine the treatment plan and predict the overall survival rate, doctors and researchers need a comprehensive prognostic evaluation system. Nomograms are being increasingly assessed in medical studies and are often used in the prediction of prognosis (Balachandran et al., 2015; Semenkovich et al., 2021). We integrated our five-gene model and other well-known clinicopathological parameters to create a nomogram. The prognosis for the children diagnosed with NB before 18 months is generally better than those diagnosed later (Vo et al., 2014). In the nomogram, age remained a crucial factor and ranked second after the five-gene model. Patients were divided into low-, medium-, and high-risk groups based on their total nomogram scores. The KM curves showed that the nomogram had excellent discriminative ability. According to the calibration curves, the predictions made by the nomogram were consistent with the observed data. The nomogram offers a visualized scoring system to facilitate medical decision making, which gives high-risk patients a chance to obtain more accurate treatments. Using it, physicians could predict the overall survival for each patient and make an individualized treatment plan. Patients in the high-risk group should be given special attention and more intensive treatment, while excessive treatment could be avoided for patients in the low–medium-risk groups.

Immunotherapy is currently a key hotspot in the field of cancer research. However, due to the difference in immunogenicity from adult tumors, targeted drugs, including immune checkpoint inhibitors, have limited applications in pediatric tumors (Wienke et al., 2021). In recent years, although new targeted therapies such as anti-GD2 therapy have exhibited great promise in treating NB, the limited efficacy and considerable toxicity make it still urgent to expand therapeutic targets and identify groups with potential benefits for NB immunotherapy (Sait and Modak, 2017; Park and Cheung, 2020). In our study, tumor immunological characteristics were assessed by the ESTIMATE algorithm. Higher immune scores and ESTIMATE scores were observed in patients with low risk scores. In light of the difference in overall survival between these two groups, more immune components in the tumor microenvironment may contribute to the better outcome of low-risk patients. In addition, the results calculated by the CIBERSORT algorithm indicated a significantly higher presence of T follicular helper cells (TFh) and activated NK cells in low-risk patients, both of which have been demonstrated to have protective roles in cancer. TFh plays an important role in humoral immunity (Eivazi et al., 2016). A previous study in NB showed that γδ TFh cells might facilitate the maturation of B cells and the production of antibodies (Mou et al., 2017). In studies of colorectal and breast cancer, infiltrating TFh cells have also been found to be positively correlated with prognosis (Bindea et al., 2013; Gu-Trantien et al., 2013). Similarly, a reduced degree of NK cell infiltration in tumor tissues has been found to be associated with a worse outcome in some patient cohorts (Albertsson et al., 2003; Mandal et al., 2016). NK cells play a central role in cancer immunotherapy (Shimasaki et al., 2020). As mentioned above, the application of anti-GD2 immunotherapy in recent years has significantly improved the survival of high-risk NB patients. NK cell-mediated antibody-dependent cellular cytotoxicity (ADCC) is a potent mechanism of anti-GD2 immunotherapy for NB. Anti-GD2 monoclonal antibodies coadministered with NK cell infusion have achieved promising results in several clinical trials. These studies demonstrated that NK cell infusion enhances ADCC directed by the antibody (Federico et al., 2017; Modak et al., 2018). Dinutuximab-mediated autologous killing of NB cells could be enhanced by plasmacytoid dendritic cell-induced NK cell activation (Belounis et al., 2020). Recent findings have shown that exosomes derived from human NK cells contain tumor suppressor miRNAs that are cytotoxic to NB cells and inhibit tumor cell immune escape, suggesting that NK cell-derived exosomes could be used as a complement to NK cell-based immunotherapy (Neviani et al., 2019). Except for these two types of cells, no differences were found between the two groups for other important immune cells in the training set, such as tumor-associated macrophages and CD8^+^ T cells. Interestingly, we obtained different results in the other two validation sets, suggesting that the different data sets selected might have been confounding. Unlike adult cancers, most pediatric solid tumors are considered immunologically cold, characterized by low tumor mutation burden, limited T-cell infiltration, and a lack of responsiveness to immune checkpoint inhibitors (Grobner et al., 2018; Wienke et al., 2021). In light of these differences between childhood tumors and adult tumors, a better understanding of NB immunobiology is needed, which will improve future immunotherapy in NB.

Among the five hub genes, AHI1, NXPH1, and CPLX3 have not been reported in any previous studies on NB. The oncogenic role of AHI1 has been identified in human leukemia and Sezary syndrome (Ringrose et al., 2006; Chen et al., 2013). Mutations in the AHI1 gene could lead to Joubert syndrome, which is a rare genetic disorder characterized by the underdevelopment of cerebellar vermis (Alvarez Retuerto et al., 2008). Moreover, it is also involved in autism (Alvarez Retuerto et al., 2008) and was considered to be a susceptibility gene for schizophrenia (Torri et al., 2010). It seems that AHI1, which has been associated with these brain developmental disorders, has a crucial role in brain growth during early life. As a specific neuronal cell surface protein, NXPH1 is essential for trans-synaptic activation (Missler et al., 2003). By using a genome-wide methylation screen, Faryna et al. found that the methylation level of NXPH1 in normal tissues was significantly higher than that in breast cancer samples, indicating it as a potential useful biomarker for tumor diagnosis (Faryna et al., 2012). In a study of intraductal papillary mucinous neoplasms (IPMNs), NXPH1 was more likely to be methylated in low-grade dysplasia than in high-grade dysplasia (Hong et al., 2012). NXPH1 was incorporated in a 10-gene signature that predicted biochemical recurrence for prostate cancer, and its expression level was upregulated in patients with a Gleason score ≥7 (Wu et al., 2020). In contrast, there was a negative correlation between lymph node metastasis and NXPH1 in pancreatic cancer (Jin and Tsai, 2016). GDPD5 is located on chromosome 11q13, and the presence of an 11q deletion is usually associated with an advanced stage and worse outcome in NB (Jiang et al., 2011). It belongs to the glycerophosphodiester phosphodiesterase family, which is critical for the metabolism of glycerol. In our study, we discovered that overexpression of GDPD5 is associated with better survival, which is in accordance with the result of a previous report in which high GDPD5 expression was found to be strongly correlated with a favorable outcome in neuroblastoma, while low GDPD5 expression was associated with a poor outcome. In addition, the study further revealed that GDPD5 induced the differentiation and inhibited the motility of NB cells via multiple mechanisms (Matas-Rico et al., 2016). GDPD5 was found to be overexpressed in colorectal cancer (CRC) and to promote metastasis and chemoresistance. Additionally, silencing GDPD5 in 5-Fu-resistant CRC cells decreased epithelial-to-mesenchymal transition (EMT) and cell invasion, both of which are essential for CRC metastasis (Feng et al., 2018). Cao et al. silenced GDPD5 in breast cancer cell lines and found a significant decrease in tumor cell viability, migration, and invasion (Cao et al., 2016). SPAG6 is identified as a cancer-testis antigen that regulates multiple functions in various cell types (Zheng et al., 2019). A variety of human cancers have been shown to be associated with SPAG6. A study on the differential analysis of genome-wide methylation of NB cell lines reported that SPAG6 was a possible target of the CpG island methylator phenotype (CIMP), and CIMP was demonstrated to be negatively related to the survival of NB patients (Abe et al., 2005; Abe et al., 2008). However, the role of SPAG6 in NB progression requires further study. CPLX3 is specifically localized to retinal ribbon synapses. It is a key regulator of transmitter release at retinal ribbon synapses (McMahon et al., 1995; Mortensen et al., 2016). At present, there is no research on CPLX3 and its role in tumors. Our results suggest that it may promote the development of NB and affect prognosis. Therefore, the specific role of CPLX3 in NB needs to be further studied. Our research has discovered five genes closely related to MYCN. Some of them have been identified to be involved in development of NB, and some have been studied in other malignant tumors. Next, we will further investigate the relationship between these five pivotal genes and MYCN as well as their roles in the development of NB.

However, there were several imitations to our study. First, since the TARGET-NBL gene expression data set used as the training set mainly contained NB cases in stage 1 and stage 4, we did not include INSS staging in the nomogram. This may limit the predictive power of the nomogram. Second, the bioinformatic analysis of this study was based on public data sets, and the tissues used for verification were retrospectively collected. Thus, an inherent bias in case selection may have influenced the findings.

## Conclusion

Overall, we developed a novel five-gene signature and successfully built a prognostic nomogram that accurately predicts the survival of NB patients. It will simplify the stratification of NB patients and provide some guidance for individualized treatment. The five hub genes obtained in this study were based on integrated analyses of multiple data sets, which provides a very high level of reliability. Prior to this study, three of the identified prognostic genes had not been associated with NB. The mechanisms of these genes and their roles in targeted therapy for NB remain to be investigated.

## Data Availability

The data sets presented in this study can be found in online repositories. The names of the repository/repositories and accession number(s) can be found in the article/Supplementary Material.

## References

[B1] Abe M. Ohira M. Kaneda A. Yagi Y. Yamamoto S. Kitano Y. (2005). CpG Island Methylator Phenotype Is a strong Determinant of Poor Prognosis in Neuroblastomas. Cancer Res. 65 (3), 828–834. doi:65/3/828[pii] 15705880

[B2] Abe M. Watanabe N. McDonell N. Takato T. Ohira M. Nakagawara A. (2008). Identification of Genes Targeted by CpG Island Methylator Phenotype in Neuroblastomas, and Their Possible Integrative Involvement in Poor Prognosis. Oncology 74 (1-2), 50–60. 10.1159/000139124 18544995

[B3] Albertsson P. A. Basse P. H. Hokland M. Goldfarb R. H. Nagelkerke J. F. Nannmark U. (2003). NK Cells and the Tumour Microenvironment: Implications for NK-Cell Function and Anti-tumour Activity. Trends Immunol. 24 (11), 603–609. 10.1016/j.it.2003.09.007 14596885

[B4] Alvarez Retuerto A. I. Cantor R. M. Gleeson J. G. Ustaszewska A. Schackwitz W. S. Pennacchio L. A. (2008). Association of Common Variants in the Joubert Syndrome Gene (AHI1) with Autism. Hum. Mol. Genet. 17 (24), 3887–3896. 10.1093/hmg/ddn291 18782849 PMC2638573

[B5] Balachandran V. P. Gonen M. Smith J. J. DeMatteo R. P. (2015). Nomograms in Oncology: More Than Meets the Eye. Lancet Oncol. 16 (4), e173–e180. 10.1016/S1470-2045(14)71116-7 25846097 PMC4465353

[B6] Belounis A. Ayoub M. Cordeiro P. Lemieux W. Teira P. Haddad E. (2020). Patients' NK Cell Stimulation with Activated Plasmacytoid Dendritic Cells Increases Dinutuximab-Induced Neuroblastoma Killing. Cancer Immunol. Immunother. 69 (9), 1767–1779. 10.1007/s00262-020-02581-0 32342128 PMC11027604

[B7] Bindea G. Mlecnik B. Tosolini M. Kirilovsky A. Waldner M. Obenauf A. C. (2013). Spatiotemporal Dynamics of Intratumoral Immune Cells Reveal the Immune Landscape in Human Cancer. Immunity 39 (4), 782–795. 10.1016/j.immuni.2013.10.003 24138885

[B8] Brodeur G. M. Seeger R. C. Schwab M. Varmus H. E. Bishop J. M. (1984). Amplification of N- Myc in Untreated Human Neuroblastomas Correlates with Advanced Disease Stage. Science 224 (4653), 1121–1124. 10.1126/science.6719137 6719137

[B9] Campbell K. Gastier-Foster J. M. Mann M. Naranjo A. H. Van Ryn C. Bagatell R. (2017). Association ofMYCNcopy Number with Clinical Features, Tumor Biology, and Outcomes in Neuroblastoma: A Report from the Children's Oncology Group. Cancer 123 (21), 4224–4235. 10.1002/cncr.30873 28696504 PMC5650521

[B10] Cao M. D. Cheng M. Rizwan A. Jiang L. Krishnamachary B. Bhujwalla Z. M. (2016). Targeting Choline Phospholipid Metabolism: GDPD5 and GDPD6 Silencing Decrease Breast Cancer Cell Proliferation, Migration, and Invasion. NMR Biomed. 29 (8), 1098–1107. 10.1002/nbm.3573 27356959 PMC5555158

[B11] Casey D. L. Cheung N.-K. V. (2020). Immunotherapy of Pediatric Solid Tumors: Treatments at a Crossroads, with an Emphasis on Antibodies. Cancer Immunol. Res. 8 (2), 161–166. 10.1158/2326-6066.CIR-19-0692 32015013 PMC7058412

[B12] Chen M. Gallipoli P. DeGeer D. Sloma I. Forrest D. L. Chan M. (2013). Targeting Primitive Chronic Myeloid Leukemia Cells by Effective Inhibition of a New AHI-1-BCR-ABL-JAK2 Complex. J. Natl. Cancer Inst. 105 (6), 405–423. 10.1093/jnci/djt006 23446755 PMC3601953

[B13] Cohn S. L. Pearson A. D. J. London W. B. Monclair T. Ambros P. F. Brodeur G. M. (2009). The International Neuroblastoma Risk Group (INRG) Classification System: an INRG Task Force Report. Jco 27 (2), 289–297. 10.1200/JCO.2008.16.6785 PMC2650388 19047291

[B14] De Preter K. Vermeulen J. Brors B. Delattre O. Eggert A. Fischer M. (2010). Accurate Outcome Prediction in Neuroblastoma across Independent Data Sets Using a Multigene Signature. Clin. Cancer Res. 16 (5), 1532–1541. 10.1158/1078-0432.CCR-09-2607 20179214

[B15] Eivazi S. Bagheri S. Hashemzadeh M. S. Ghalavand M. Qamsari E. S. Dorostkar R. (2016). Development of T Follicular Helper Cells and Their Role in Disease and Immune System. Biomed. Pharmacother. 84, 1668–1678. 10.1016/j.biopha.2016.10.083 27863842

[B16] Faryna M. Konermann C. Aulmann S. Bermejo J. L. Brugger M. Diederichs S. (2012). Genome‐wide Methylation Screen in Low‐grade Breast Cancer Identifies Novel Epigenetically Altered Genes as Potential Biomarkers for Tumor Diagnosis. FASEB j. 26 (12), 4937–4950. 10.1096/fj.12-209502 22930747

[B17] Federico S. M. McCarville M. B. Shulkin B. L. Sondel P. M. Hank J. A. Hutson P. (2017). A Pilot Trial of Humanized Anti-GD2 Monoclonal Antibody (hu14.18K322A) with Chemotherapy and Natural Killer Cells in Children with Recurrent/Refractory Neuroblastoma. Clin. Cancer Res. 23 (21), 6441–6449. 10.1158/1078-0432.CCR-17-0379 28939747 PMC8725652

[B18] Feng C. Zhang L. Sun Y. Li X. Zhan L. Lou Y. (2018). GDPD5, a Target of miR-195-5p, Is Associated with Metastasis and Chemoresistance in Colorectal Cancer. Biomed. Pharmacother. 101, 945–952. 10.1016/j.biopha.2018.03.028 29635904

[B19] Gröbner S. N. Worst B. C. Weischenfeldt J. Buchhalter I. Kleinheinz K. Rudneva V. A. (2018). The Landscape of Genomic Alterations across Childhood Cancers. Nature 555 (7696), 321–327. 10.1038/nature25480 29489754

[B20] Gu-Trantien C. Loi S. Garaud S. Equeter C. Libin M. de Wind A. (2013). CD4+ Follicular Helper T Cell Infiltration Predicts Breast Cancer Survival. J. Clin. Invest. 123 (7), 2873–2892. 10.1172/JCI67428 23778140 PMC3696556

[B21] Gunda V. Pathania A. S. Chava S. Prathipati P. Chaturvedi N. K. Coulter D. W. (2020). Amino Acids Regulate Cisplatin Insensitivity in Neuroblastoma. Cancers 12 (9), 2576. 10.3390/cancers12092576 PMC7563727 32927667

[B22] Gustafson W. C. Meyerowitz J. G. Nekritz E. A. Chen J. Benes C. Charron E. (2014). Drugging MYCN through an Allosteric Transition in Aurora Kinase A. Cancer Cell 26 (3), 414–427. 10.1016/j.ccr.2014.07.015 25175806 PMC4160413

[B23] He X. Qin C. Zhao Y. Zou L. Zhao H. Cheng C. (2020). Gene Signatures Associated with Genomic Aberrations Predict Prognosis in Neuroblastoma. Cancer Commun. 40 (2-3), 105–118. 10.1002/cac2.12016 PMC7163660 32237073

[B24] Hong S.-M. Omura N. Vincent A. Li A. Knight S. Yu J. (2012). Genome-wide CpG Island Profiling of Intraductal Papillary Mucinous Neoplasms of the Pancreas. Clin. Cancer Res. 18 (3), 700–712. 10.1158/1078-0432.CCR-11-1718 22173550 PMC3271174

[B25] Huang C.-T. Hsieh C.-H. Lee W.-C. Liu Y.-L. Yang T.-S. Hsu W.-M. (2019). Therapeutic Targeting of Non-oncogene Dependencies in High-Risk Neuroblastoma. Clin. Cancer Res. 25 (13), 4063–4078. 10.1158/1078-0432.CCR-18-4117 30952635

[B26] Huang M. Weiss W. A. (2013). Neuroblastoma and MYCN. Cold Spring Harbor Perspect. Med. 3 (10), a014415. 10.1101/cshperspect.a014415 PMC3784814 24086065

[B27] Janoueix-Lerosey I. Schleiermacher G. Michels E. Mosseri V. Ribeiro A. Lequin D. (2009). Overall Genomic Pattern Is a Predictor of Outcome in Neuroblastoma. Jco 27 (7), 1026–1033. 10.1200/JCO.2008.16.0630 19171713

[B28] Jiang M. Stanke J. Lahti J. M. (2011). The Connections between Neural Crest Development and Neuroblastoma. Curr. Top. Dev. Biol. 94, 77–127. 10.1016/B978-0-12-380916-2.00004-8 21295685 PMC3633592

[B29] Jin J.-S. Tsai W. C. (2016). The Detection of Tumor Location and Lymph Node Metastasis by Aberrant NXPH1 and NXPH2 Expressions in Pancreatic Ductal Adenocarcinomas. Chin. J. Physiol. 59 (6), 348–354. 10.4077/CJP.2016.BAF430 27817196

[B30] Kawano A. Hazard F. K. Chiu B. Naranjo A. LaBarre B. London W. B. (2021). Stage 4S Neuroblastoma. Am. J. Surg. Pathol. 45 (8), 1075–1081. 10.1097/PAS.0000000000001647 33739795 PMC8217390

[B31] Kolde R. Laur S. Adler P. Vilo J. (2012). Robust Rank Aggregation for Gene List Integration and Meta-Analysis. Bioinformatics 28 (4), 573–580. 10.1093/bioinformatics/btr709 22247279 PMC3278763

[B32] Li J. Thompson T. D. Miller J. W. Pollack L. A. Stewart S. L. (2008). Cancer Incidence Among Children and Adolescents in the United States, 2001-2003. Pediatrics 121 (6), e1470–e1477. 10.1542/peds.2007-2964 18519450

[B33] Mandal R. Şenbabaoğlu Y. Desrichard A. Havel J. J. Dalin M. G. Riaz N. (2016). The Head and Neck Cancer Immune Landscape and its Immunotherapeutic Implications. JCI Insight 1 (17), e89829. 10.1172/jci.insight.89829 27777979 PMC5070962

[B34] Maris J. M. (2010). Recent Advances in Neuroblastoma. N. Engl. J. Med. 362 (23), 2202–2211. 10.1056/NEJMra0804577 20558371 PMC3306838

[B35] Marshall G. M. Carter D. R. Cheung B. B. Liu T. Mateos M. K. Meyerowitz J. G. (2014). The Prenatal Origins of Cancer. Nat. Rev. Cancer 14 (4), 277–289. 10.1038/nrc3679 24599217 PMC4041218

[B36] Martin T. A. Jiang W. G. (2009). Loss of Tight junction Barrier Function and its Role in Cancer Metastasis. Biochim. Biophys. Acta (Bba) - Biomembranes 1788 (4), 872–891. 10.1016/j.bbamem.2008.11.005 19059202

[B37] Matas-Rico E. van Veen M. Leyton-Puig D. van den Berg J. Koster J. Kedziora K. M. (2016). Glycerophosphodiesterase GDE2 Promotes Neuroblastoma Differentiation through Glypican Release and Is a Marker of Clinical Outcome. Cancer Cell 30 (4), 548–562. 10.1016/j.ccell.2016.08.016 27693046

[B38] McMahon H. T. Missler M. Li C. Südhof T. C. (1995). Complexins: Cytosolic Proteins that Regulate SNAP Receptor Function. Cell 83 (1), 111–119. 10.1016/0092-8674(95)90239-2 7553862

[B39] Missler M. Zhang W. Rohlmann A. Kattenstroth G. Hammer R. E. Gottmann K. (2003). α-Neurexins Couple Ca2+ Channels to Synaptic Vesicle Exocytosis. Nature 423 (6943), 939–948. 10.1038/nature01755 12827191

[B40] Modak S. Le Luduec J.-B. Cheung I. Y. Goldman D. A. Ostrovnaya I. Doubrovina E. (2018). Adoptive Immunotherapy with Haploidentical Natural Killer Cells and Anti-GD2 Monoclonal Antibody m3F8 for Resistant Neuroblastoma: Results of a Phase I Study. Oncoimmunology 7 (8), e1461305. 10.1080/2162402X.2018.1461305 30221057 PMC6136849

[B41] Mortensen L. S. Park S. J. H. Ke J.-b. Cooper B. H. Zhang L. Imig C. (2016). Complexin 3 Increases the Fidelity of Signaling in a Retinal Circuit by Regulating Exocytosis at Ribbon Synapses. Cel Rep. 15 (10), 2239–2250. 10.1016/j.celrep.2016.05.012 PMC5134263 27239031

[B42] Mou W. Han W. Ma X. Wang X. Qin H. Zhao W. (2017). γδTFH Cells Promote B Cell Maturation and Antibody Production in Neuroblastoma. BMC Immunol. 18 (1), 36. 10.1186/s12865-017-0216-x 28687069 PMC5500960

[B43] Neviani P. Wise P. M. Murtadha M. Liu C. W. Wu C.-H. Jong A. Y. (2019). Natural Killer-Derived Exosomal miR-186 Inhibits Neuroblastoma Growth and Immune Escape Mechanisms. Cancer Res. 79 (6), 1151–1164. 10.1158/0008-5472.CAN-18-0779 30541743 PMC6428417

[B44] Newman A. M. Liu C. L. Green M. R. Gentles A. J. Feng W. Xu Y. (2015). Robust Enumeration of Cell Subsets from Tissue Expression Profiles. Nat. Methods 12 (5), 453–457. 10.1038/nmeth.3337 25822800 PMC4739640

[B45] Park J. A. Cheung N.-K. V. (2020). Targets and Antibody Formats for Immunotherapy of Neuroblastoma. Jco 38 (16), 1836–1848. 10.1200/JCO.19.01410 PMC7255979 32167865

[B46] Park J. R. Bagatell R. London W. B. Maris J. M. Cohn S. L. Mattay K. M. (2013). Children's Oncology Group's 2013 Blueprint for Research: Neuroblastoma. Pediatr. Blood Cancer 60 (6), 985–993. 10.1002/pbc.24433 23255319

[B47] Pinto N. R. Applebaum M. A. Volchenboum S. L. Matthay K. K. London W. B. Ambros P. F. (2015). Advances in Risk Classification and Treatment Strategies for Neuroblastoma. Jco 33 (27), 3008–3017. 10.1200/JCO.2014.59.4648 PMC4567703 26304901

[B48] Puissant A. Frumm S. M. Alexe G. Bassil C. F. Qi J. Chanthery Y. H. (2013). Targeting MYCN in Neuroblastoma by BET Bromodomain Inhibition. Cancer Discov. 3 (3), 308–323. 10.1158/2159-8290.CD-12-0418 23430699 PMC3672953

[B49] Ringrose A. Zhou Y. Pang E. Zhou L. Lin A. E.-J. Sheng G. (2006). Evidence for an Oncogenic Role of AHI-1 in Sezary Syndrome, a Leukemic Variant of Human Cutaneous T-Cell Lymphomas. Leukemia 20 (9), 1593–1601. 10.1038/sj.leu.2404321 16838023

[B50] Ritchie M. E. Phipson B. Wu D. Hu Y. Law C. W. Shi W. (2015). Limma powers Differential Expression Analyses for RNA-Sequencing and Microarray Studies. Nucleic Acids Res. 43 (7), e47. 10.1093/nar/gkv007 25605792 PMC4402510

[B51] Sait S. Modak S. (2017). Anti-GD2 Immunotherapy for Neuroblastoma. Expert Rev. Anticancer Ther. 17 (10), 889–904. 10.1080/14737140.2017.1364995 28780888 PMC6082365

[B52] Schleiermacher G. Mosseri V. London W. B. Maris J. M. Brodeur G. M. Attiyeh E. (2012). Segmental Chromosomal Alterations Have Prognostic Impact in Neuroblastoma: a Report from the INRG Project. Br. J. Cancer 107 (8), 1418–1422. 10.1038/bjc.2012.375 22976801 PMC3494425

[B53] Seeger R. C. Brodeur G. M. Sather H. Dalton A. Siegel S. E. Wong K. Y. (1985). Association of Multiple Copies of the N-mycOncogene with Rapid Progression of Neuroblastomas. N. Engl. J. Med. 313 (18), 1111–1116. 10.1056/NEJM198510313131802 4047115

[B54] Semenkovich T. R. Yan Y. Subramanian M. Meyers B. F. Kozower B. D. Nava R. (2021). A Clinical Nomogram for Predicting Node-Positive Disease in Esophageal Cancer. Ann. Surg. 273 (6), e214–e221. 10.1097/SLA.0000000000003450 31274650 PMC6940556

[B55] Shimasaki N. Jain A. Campana D. (2020). NK Cells for Cancer Immunotherapy. Nat. Rev. Drug Discov. 19 (3), 200–218. 10.1038/s41573-019-0052-1 31907401

[B56] Smoot M. E. Ono K. Ruscheinski J. Wang P.-L. Ideker T. (2011). Cytoscape 2.8: New Features for Data Integration and Network Visualization. Bioinformatics 27 (3), 431–432. 10.1093/bioinformatics/btq675 21149340 PMC3031041

[B57] Suzuki M. Cheung N.-K. V. (2015). Disialoganglioside GD2 as a Therapeutic Target for Human Diseases. Expert Opin. Ther. Targets 19 (3), 349–362. 10.1517/14728222.2014.986459 25604432

[B58] Szklarczyk D. Gable A. L. Lyon D. Junge A. Wyder S. Huerta-Cepas J. (2019). STRING V11: Protein-Protein Association Networks with Increased Coverage, Supporting Functional Discovery in Genome-wide Experimental Datasets. Nucleic Acids Res. 47 (D1), D607–D613. 10.1093/nar/gky1131 30476243 PMC6323986

[B59] Torri F. Akelai A. Lupoli S. Sironi M. Amann‐Zalcenstein D. Fumagalli M. (2010). Fine Mapping ofAHI1as a Schizophrenia Susceptibility Gene: from Association to Evolutionary Evidence. FASEB j. 24 (8), 3066–3082. 10.1096/fj.09-152611 20371615

[B60] Vermeulen J. De Preter K. Naranjo A. Vercruysse L. Van Roy N. Hellemans J. (2009). Predicting Outcomes for Children with Neuroblastoma Using a Multigene-Expression Signature: a Retrospective SIOPEN/COG/GPOH Study. Lancet Oncol. 10 (7), 663–671. 10.1016/S1470-2045(09)70154-8 19515614 PMC3045079

[B61] Vettore L. Westbrook R. L. Tennant D. A. (2020). New Aspects of Amino Acid Metabolism in Cancer. Br. J. Cancer 122 (2), 150–156. 10.1038/s41416-019-0620-5 31819187 PMC7052246

[B62] Vo K. T. Matthay K. K. Neuhaus J. London W. B. Hero B. Ambros P. F. (2014). Clinical, Biologic, and Prognostic Differences on the Basis of Primary Tumor Site in Neuroblastoma: a Report from the International Neuroblastoma Risk Group Project. Jco 32 (28), 3169–3176. 10.1200/JCO.2014.56.1621 PMC4171360 25154816

[B63] Wang Z. Cheng H. Xu H. Yu X. Sui D. (2020). A Five-Gene Signature Derived from m6A Regulators to Improve Prognosis Prediction of Neuroblastoma. Cbm 28 (3), 275–284. 10.3233/CBM-191196 PMC12662367 32176634

[B64] Wienke J. Dierselhuis M. P. Tytgat G. A. M. Künkele A. Nierkens S. Molenaar J. J. (2021). The Immune Landscape of Neuroblastoma: Challenges and Opportunities for Novel Therapeutic Strategies in Pediatric Oncology. Eur. J. Cancer 144, 123–150. 10.1016/j.ejca.2020.11.014 33341446

[B68] Wu X. Lv D. Lei M. Cai C. Zhao Z. Eftekhar M. (2020). A 10-Gene Signature as a Predictor of Biochemical Recurrence After Radical Prostatectomy in Patients With Prostate Cancer and a Gleason Score ≥7. Oncol. Lett. 20 (3), 2906–2918. 10.3892/ol.2020.11830 32782607 PMC7400999

[B65] Yoshihara K. Shahmoradgoli M. Martínez E. Vegesna R. Kim H. Torres-Garcia W. (2013). Inferring Tumour Purity and Stromal and Immune Cell Admixture from Expression Data. Nat. Commun. 4, 2612. 10.1038/ncomms3612 24113773 PMC3826632

[B66] Yu G. Wang L.-G. Han Y. He Q.-Y. (2012). clusterProfiler: an R Package for Comparing Biological Themes Among Gene Clusters. OMICS: A J. Integr. Biol. 16 (5), 284–287. 10.1089/omi.2011.0118 PMC3339379 22455463

[B67] Zheng D.-F. Wang Q. Wang J.-P. Bao Z.-Q. Wu S.-W. Ma L. (2019). The Emerging Role of Sperm-Associated Antigen 6 Gene in the Microtubule Function of Cells and Cancer. Mol. Ther. - Oncolytics 15, 101–107. 10.1016/j.omto.2019.08.011 31660426 PMC6807308

