# Supplementary figures and images for: Identification of a Five-Gene Signature Derived From MYCN Amplification and Establishment of a Nomogram for Predicting the Prognosis of Neuroblastoma
Source: Front Mol Biosci. 2021 Dec 7;8:769661. doi: 10.3389/fmolb.2021.769661 (PMC8691574; doi:10.3389/fmolb.2021.769661)

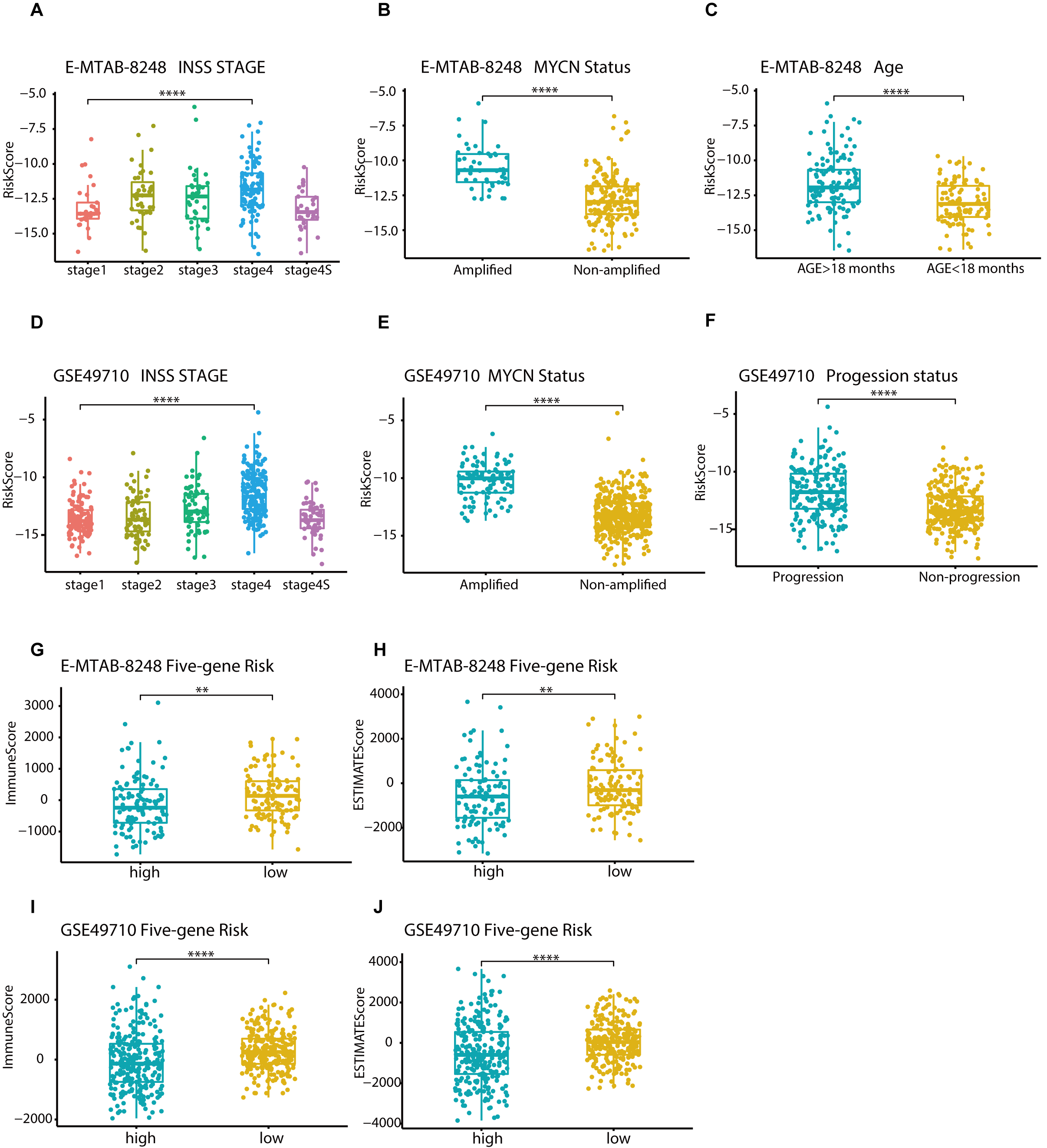

Supplement: Supplementary file 2 [file Image6.TIF]

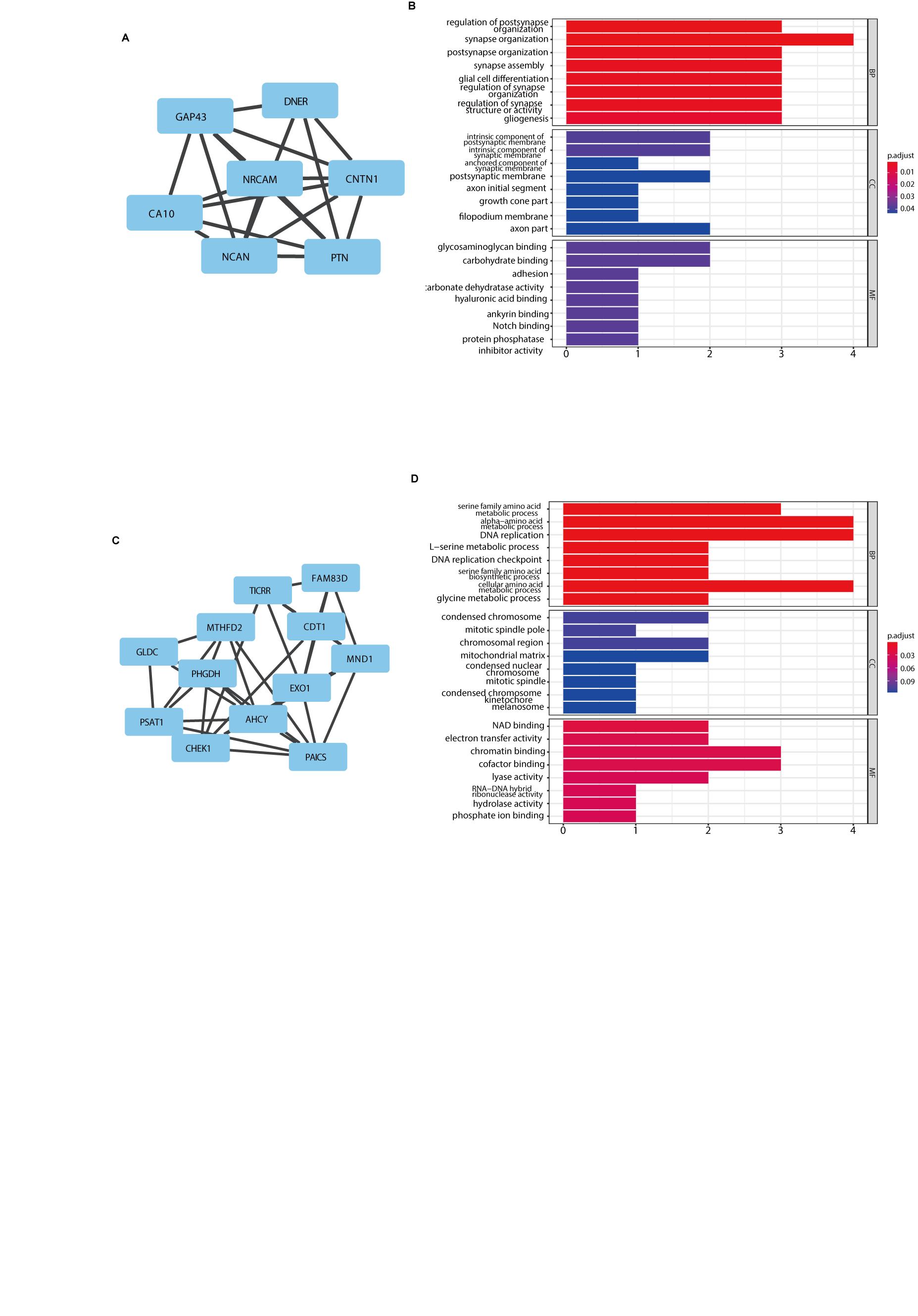

Supplement: Supplementary file 4 [file Image3.TIF]

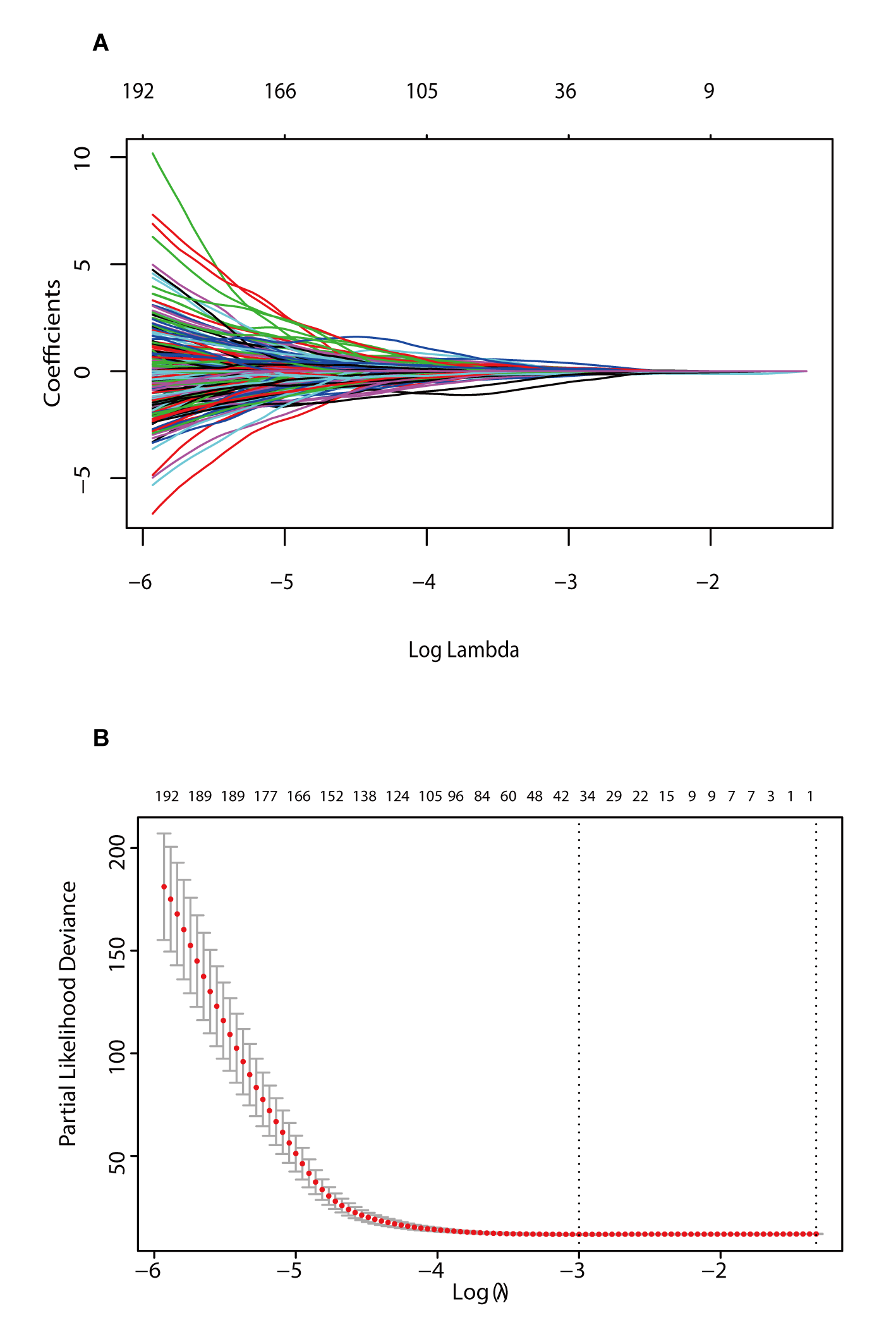

Supplement: Supplementary file 5 [file Image4.TIF]

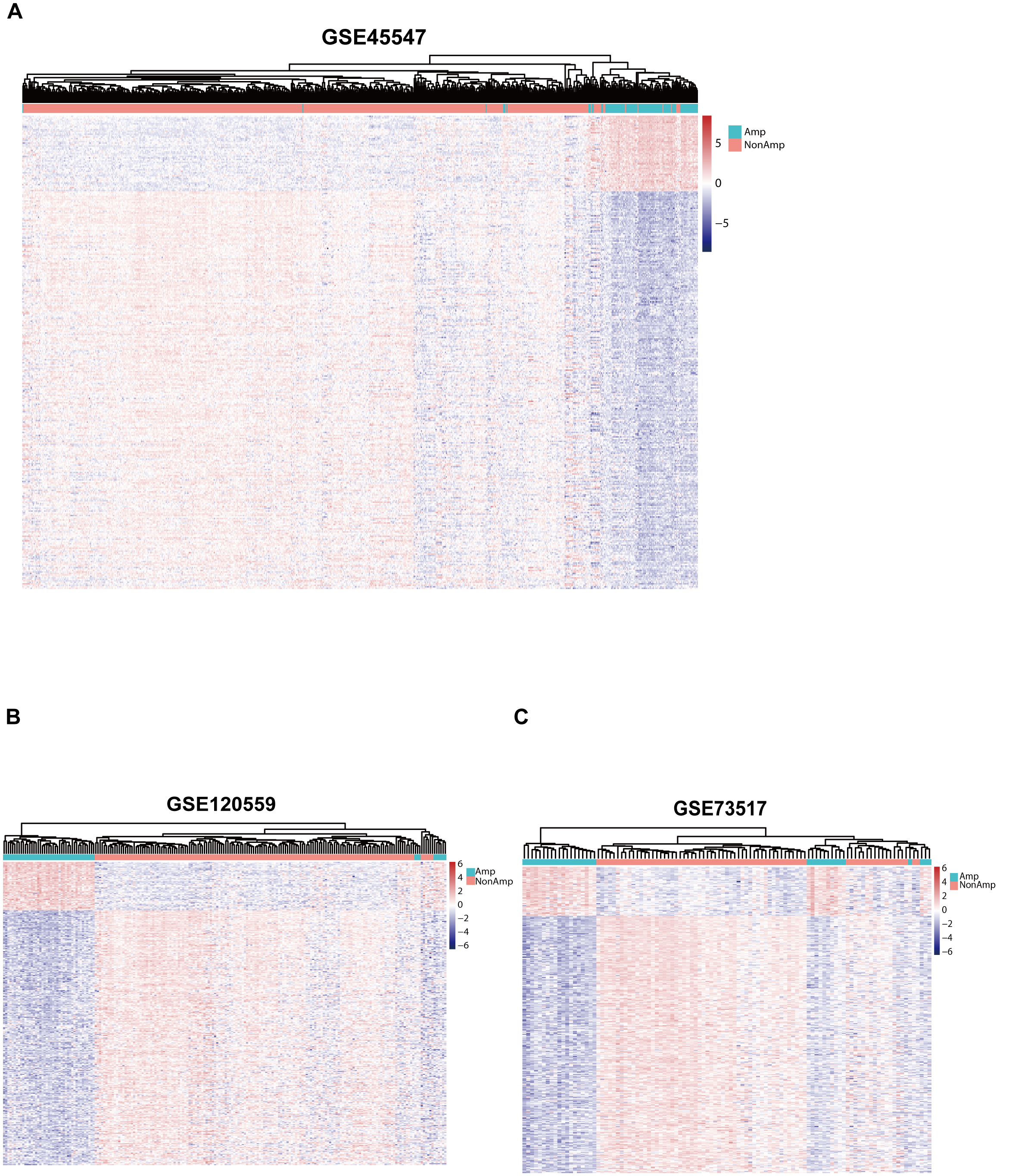

Supplement: Supplementary file 6 [file Image2.TIF]

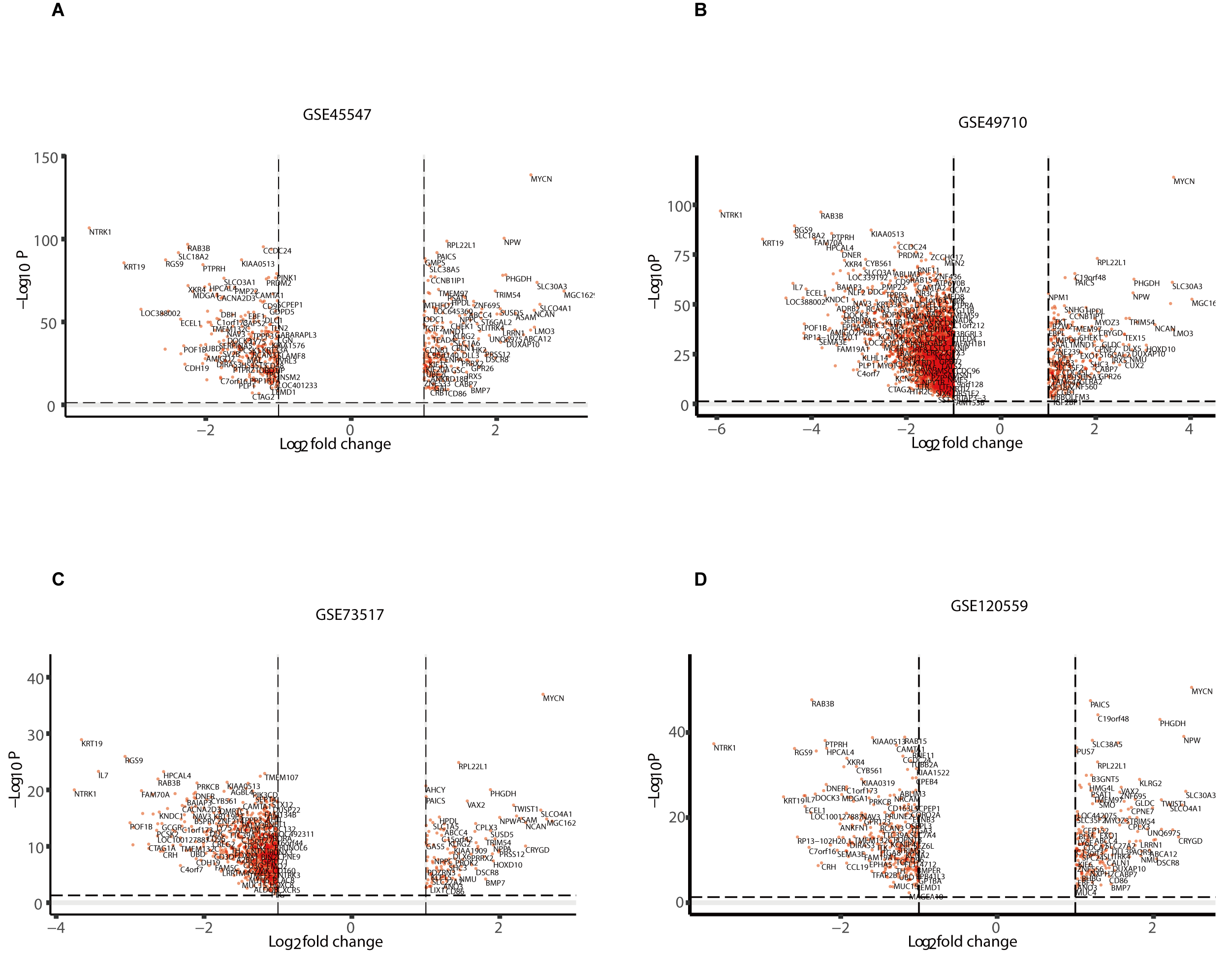

Supplement: Supplementary file 7 [file Image1.TIF]

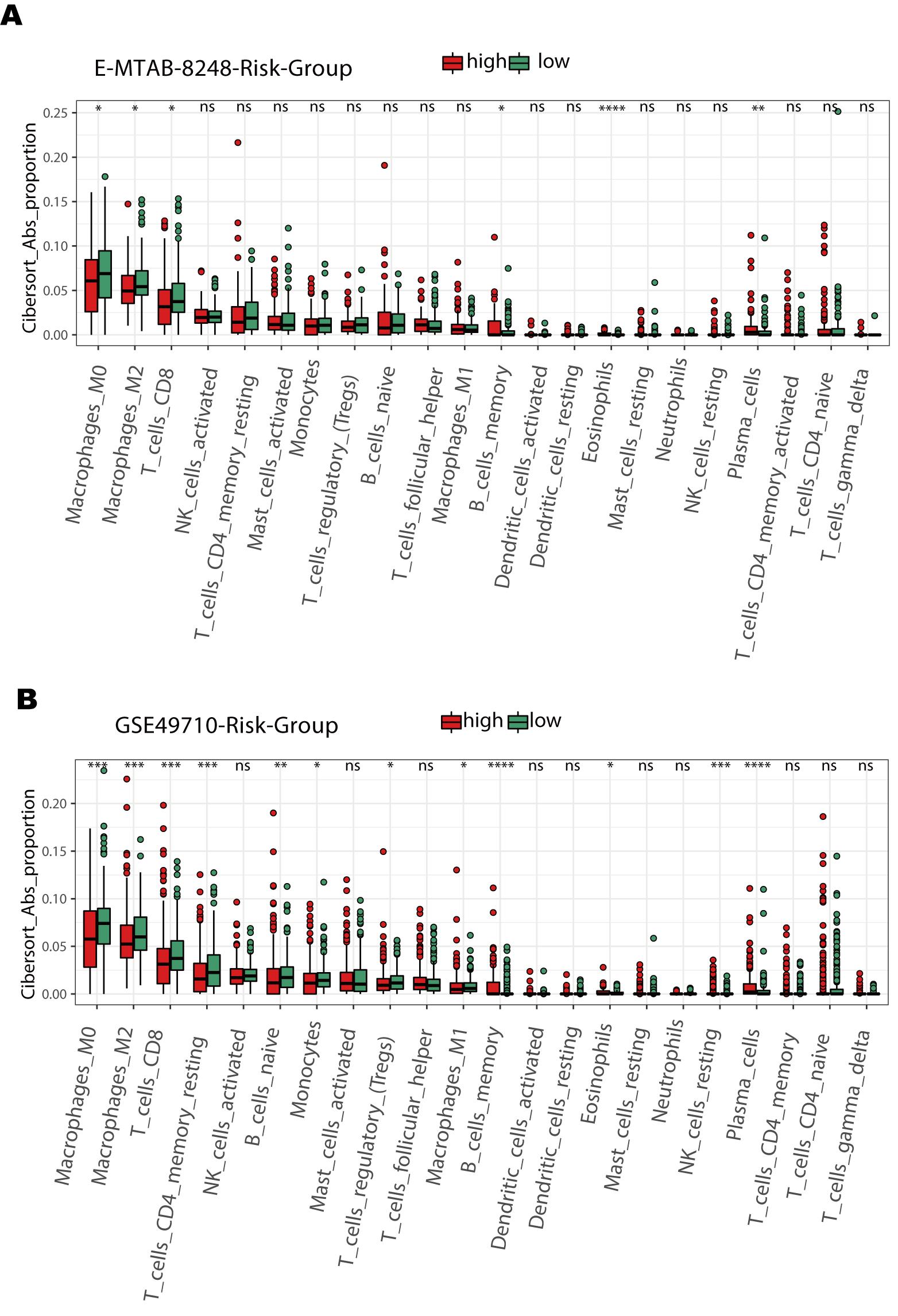

Supplement: Supplementary file 8 [file Image7.TIF]

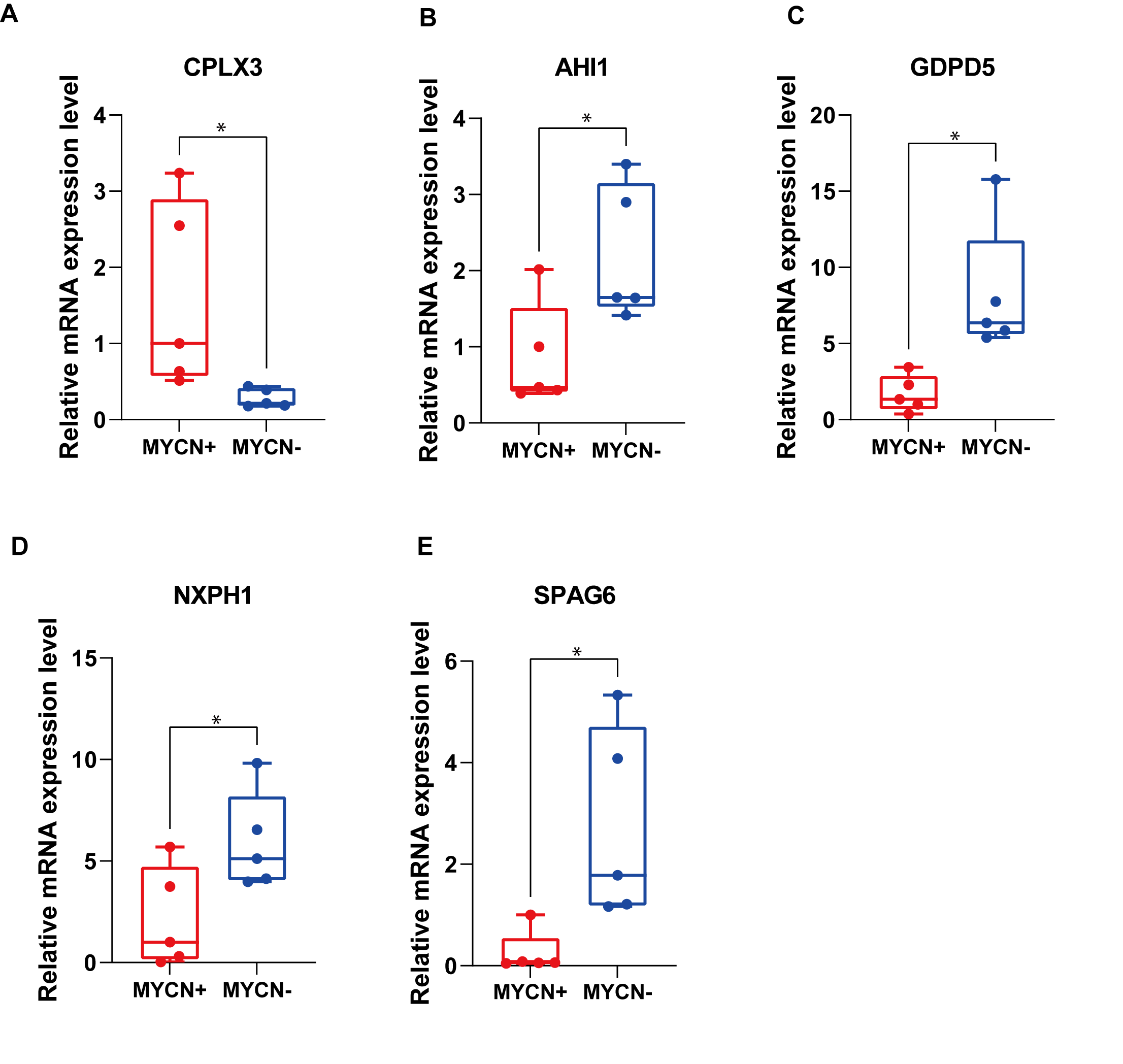

Supplement: Supplementary file 11 [file Image8.TIF]

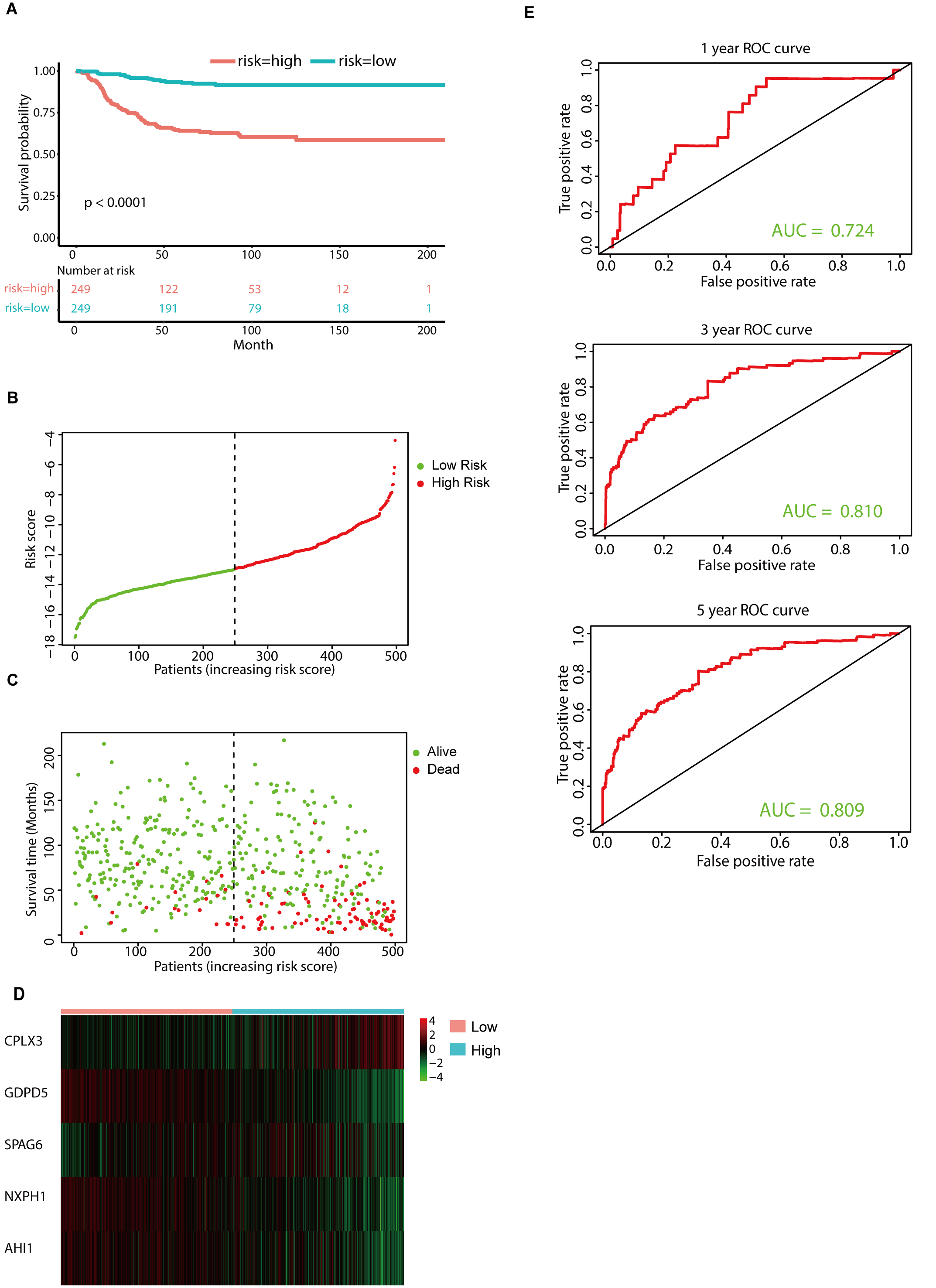

Supplement: Supplementary file 13 [file Image5.TIF]
