# Supplementary material for: Identification of a Five-Gene Signature Derived From MYCN Amplification and Establishment of a Nomogram for Predicting the Prognosis of Neuroblastoma
Source: Front Mol Biosci. 2021 Dec 7;8:769661. doi: 10.3389/fmolb.2021.769661 (PMC8691574; doi:10.3389/fmolb.2021.769661)
Supplement: Supplementary file 9 [file Table2.DOCX]

**Supplementary Table 2 Details of the Datasets Included in This Study.**

| **Datasets** | **SOURCE and Platform** | **Sample Size** | **Application** |
| --- | --- | --- | --- |
| GSE45547 | GEO, Agilent-020382 Human Custom Microarray 44k | 649 | Identiﬁcation of DEGs |
| GSE49710 | GEO, Agilent-020382 Human Custom Microarray 44k | 498 | Identiﬁcation of DEGs and validation |
| GSE73517 | GEO, Agilent-020382 Human Custom Microarray 44k | 105 | Identiﬁcation of DEGs |
| GSE120559 | GEO, Agilent-020382 Human Custom Microarray 44k | 208 | Identiﬁcation of DEGs |
| E-MTAB-8248 | ArrayExpress, Agilent-020382 Human Custom Microarray 44k | 223 | Validation |
| TARGET | NCI,Affymetrix Human Exon ST Array | 247 | Identiﬁcation of OS releated gene and model construction |
