# Supplementary material for: Identification of a Five-Gene Signature Derived From MYCN Amplification and Establishment of a Nomogram for Predicting the Prognosis of Neuroblastoma
Source: Front Mol Biosci. 2021 Dec 7;8:769661. doi: 10.3389/fmolb.2021.769661 (PMC8691574; doi:10.3389/fmolb.2021.769661)
Supplement: Supplementary file 12 [file Table6.DOCX]

**Supplementary Table 6 Clinical Characteristics of NB Patients in the TARGET NBL Dataset**

| **Clinical features** | **level** | **Overall(Mean + SD)** |
| --- | --- | --- |
| AGE (year) |  | 3.63 (2.09) |
| INSS stage (%) | Stage 1 | 30 (12.1) |
|  | Stage 3 | 1 (0.4) |
|  | Stage 4 | 216 (87.4) |
| GRADE (%) | Differentiating | 13 (5.3) |
|  | Undifferentiated or Poorly Differentiated | 170 (68.8) |
|  | Unknown | 64 (25.9) |
| Gender (%) | Female | 105 (42.5) |
|  | Male | 142 (57.5) |
| MYCN status(%) | Amplified | 68 (27.5) |
|  | Not Amplified | 175 (70.9) |
|  | Unknown | 4 (1.6) |
| Ploidy value (%) | Diploid (DI=1) | 63 (25.5) |
|  | Hyperdiploid (DI>1) | 104 (42.1) |
|  | Unknown | 80 (32.4) |
| RACE (%) | American Indian or Alaska Native | 1 (0.4) |
|  | Asian | 3 (1.2) |
|  | Black or African American | 31 (12.6) |
|  | Native Hawaiian or other Pacific Islander | 3 (1.2) |
|  | Not Reported | 8 (3.2) |
|  | Unknown | 22 (8.9) |
|  | White | 179 (72.5) |
| MKI (%) | High | 52 (21.1) |
|  | Intermediate | 55 (22.3) |
|  | Low | 69 (27.9) |
|  | Unknown | 71 (28.7) |

Abbreviations: SD, standard deviation; INSS stage, International Neuroblastoma Staging System Stage; MKI, Mitosis–Karyorrhexis Index.
