# Supplementary material for: Identification of a Five-Gene Signature Derived From MYCN Amplification and Establishment of a Nomogram for Predicting the Prognosis of Neuroblastoma
Source: Front Mol Biosci. 2021 Dec 7;8:769661. doi: 10.3389/fmolb.2021.769661 (PMC8691574; doi:10.3389/fmolb.2021.769661)
Supplement: Supplementary file 17 [file DataSheet1.DOCX]

**Figure Legends**

**Supplementary Figure 1** | **Volcano plot of DEGs in GSE45547, GSE49710, GSE73517 and GSE120559 datasets.** Only significantly changed gene were displayed.

**Supplementary Figure 2** | **DEGs between the MYCN amplification and nonamplification groups**. **(A-C)** Heatmap of the 319 DEGs after integrated analysis in the GSE45547, GSE120559 and GSE73517 dataset.

**Supplementary Figure 3** | **PPI network analysis of the DEGs. (A-B)** Clustering module 1 calculated by MCODE plugin in cytoscaple with a score of 6.333 and its GO enrichment results. **(C-D)** Clustering module 2 calculated by MCODE plugin in cytoscaple with a score of 5.818 and its GO enrichment results.

**Supplementary Figure 4** | **Thirty-five DEGs correlated with survival identified by LASSO-penalized Cox regression analysis** (A)The predictors coefficients scores as a function of lambda (λ) indicating the shrinkage of coefficients for larger numbers of log(λ). The top numbering of the plot indicates the number of predictors (variables) the model is using, going from all predictors to more sparse models. (B)The 10-fold cross-validated mean squared error as a function of (log) lambda (λ) for the lasso regularized model using the full data with interaction terms. The top numbering of the plot indicates the number of predictors (variables) the model is using, going from all predictors to more sparse models.

**Supplementary Figure 5** | **External validation of the five-gene signature. (A)** KM curves for overall survival of the two risk groups derived from the five-gene signature in the GSE49710 dataset. The p-value was calculated by the log-rank test. **(B-D)** Distribution of the risk score (B), the associated survival data (C), and the five-gene mRNA expression (D) in the GSE49710 dataset. **(E)** ROC curves for 1-year, 3-year, and 5-year overall survival predictions for the five-gene signature in the E- GSE49710 dataset.

**Supplementary Figure 6** | **Association of the five-gene signature with clinicopathological parameters in external datasets.** **(A)** Show the distribution of the risk score in different INSS stage in E-MTAB-8248 datasets. **(B)** Show the distribution of the risk score in different MYCN status in E-MTAB-8248 datasets. **(C)** Show the distribution of the risk score in different age group in E-MTAB-8248datasets. **(D)** Show the distribution of the risk score in different INSS stage in GSE49710 datasets. **(E)** Show the distribution of the risk score in different MYCN status in GSE49710 datasets. **(F)** Show the distribution of the risk score in different progression status group in GSE49710 datasets. **(G-H)** Show the distribution of the immune score and ESTIMATE score in high risk and low risk group in E-MTAB-8248 dataset. Scores were calculated with the ESTIMATE algorithm in R. **(I-J)** Show the distribution of the immune score and ESTIMATE score in high risk and low risk group in GSE49710 dataset. Scores were calculated with the ESTIMATE algorithm in R. **p < 0.01, ****p < 0.0001, two-sided unpaired Wilcoxon test.

**Supplementary Figure 7** | **Association of the five-gene signature with tumor immune components.**

**(A)** Box plots of the distribution of the cell proportions calculated by the CIBERSORT algorithm of immune cells between the high-risk and low-risk groups in E-MTAB-8248 dataset. *p < 0.05, **p < 0.01, ****p < 0.0001, n.s., not signiﬁcant, two-sided unpaired Wilcoxon test. **(B)** Box plots of the distribution of the cell proportions calculated by the CIBERSORT algorithm of immune cells between the high-risk and low-risk groups in GSE49710 dataset. *p < 0.05, **p < 0.01, ***p < 0.001, ****p < 0.0001, n.s., not signiﬁcant, two-sided unpaired Wilcoxon test.

**Supplementary Figure 8** | **Validation of the relationship between the five genes and MYCN amplification status (A-E)** Relative mRNA expression level of CPLX3, AHI1, GDPD5, NXPH1 and SPAG6 in MYCN-amplified and MYCN-nonamplified NB tissues, *p < 0.05.
